# Mouse embryonic stem cell-derived blood–brain barrier model: applicability to studying antibody triggered receptor mediated transcytosis

**DOI:** 10.1186/s12987-023-00437-0

**Published:** 2023-05-26

**Authors:** Anna Jezierski, Jez Huang, Arsalan S. Haqqani, Julie Haukenfrers, Ziying Liu, Ewa Baumann, Caroline Sodja, Claudie Charlebois, Christie E. Delaney, Alexandra T. Star, Qing Liu, Danica B. Stanimirovic

**Affiliations:** 1 Human Health Therapeutics Research Centre, National Research Council of Canada, ON Ottawa, Canada; 2 Department of Biochemistry, Microbiology and Immunology, Faculty of Medicine, University of Ottawa, Ottawa, ON Canada

**Keywords:** Blood brain barrier, Mouse brain endothelial cells, Receptor mediated transcytosis, Antibodies, Apparent permeability, Embryonic stem cells

## Abstract

Blood brain barrier (BBB) models in vitro are an important tool to aid in the pre-clinical evaluation and selection of BBB-crossing therapeutics. Stem cell derived BBB models have recently demonstrated a substantial advantage over primary and immortalized brain endothelial cells (BECs) for BBB modeling. Coupled with recent discoveries highlighting significant species differences in the expression and function of key BBB transporters, the field is in need of robust, species-specific BBB models for improved translational predictability. We have developed a mouse BBB model, composed of mouse embryonic stem cell (mESC-D3)-derived brain endothelial-like cells (mBECs), employing a directed monolayer differentiation strategy. Although the mBECs showed a mixed endothelial-epithelial phenotype, they exhibited high transendothelial electrical resistance, inducible by retinoic acid treatment up to 400 Ω cm^2^. This tight cell barrier resulted in restricted sodium fluorescein permeability (1.7 × 10^–5^ cm/min), significantly lower than that of bEnd.3 cells (1.02 × 10^–3^ cm/min) and comparable to human induced pluripotent stem cell (iPSC)-derived BECs (2.0 × 10^–5^ cm/min). The mBECs expressed tight junction proteins, polarized and functional P-gp efflux transporter and receptor mediated transcytosis (RMT) receptors; collectively important criteria for studying barrier regulation and drug delivery applications in the CNS. In this study, we compared transport of a panel of antibodies binding species selective or cross-reactive epitopes on BBB RMT receptors in both the mBEC and human iPSC-derived BEC model, to demonstrate discrimination of species-specific BBB transport mechanisms.

**Supplementary Information:**

The online version contains supplementary material available at 10.1186/s12987-023-00437-0.

## Introduction

The blood–brain barrier (BBB) is a protective barrier between the blood and brain formed by non-fenestrated brain endothelial cells (BECs). The BECs are characterized by high transendothelial electrical resistance (TEER), low permeability and vesicular transport, and high expression of tight junction proteins important for maintaining the physical barrier. In addition, efflux transporters, such as P-glycoprotein (P-gp), contribute to barrier properties by eliminating small lipophilic molecules that diffuse into BECs back into the bloodstream. BECs are also endowed with a network of specific influx transport systems to shuttle essential nutrients and metabolites across the BBB. Due to this specialized role, the BBB also prevents uptake of most small-molecule and biologic pharmaceuticals delivered intravenously, hampering the development of drugs for neurological diseases. The development of more effective neuropharmaceuticals that can cross the BBB requires a better understanding of the expression and functionality of transporters in the human and rodent BBB since rodents are typically used in preclinical assays.

Ligands or antibodies targeting BBB-enriched receptors, that undergo receptor mediated transcytosis (RMT) across the brain endothelium, are being developed to deliver therapeutic cargos into the brain. RMT receptors such transferrin receptor (TfR), insulin receptor (IR), insulin-like growth factor 1 receptor (IGF1R), low-density lipoprotein receptor (LDLR) and LDL-related protein 1 (LRP1) receptor exhibit differential expression/abundance in BEC of different species [1–5]. Antibodies developed against these receptors often show binding to species-selective epitopes, such as some antibodies developed for TfR [6], necessitating the development of ‘humanized’ mouse models expressing human extracellular domains of these receptors. These issues compound translational development of antibody-based BBB carriers in pre-clinical models. To accelerate pre-clinical screening of BBB-enabled central nervous system (CNS) targeting pipelines, it would be advantageous to develop BBB models in vitro from different species, notably mouse and human.

BBB models in vitro are routinely used to aid in the preclinical evaluation and selection of CNS targeting therapeutics. Although significant and important progress has been made in the last decade using human induced pluripotent stem cells (iPSCs) to develop human BBB models with improved scalability, high transendothelial electrical resistance (TEER), barrier-like transporter activity and potential to generate syngeneic cultures of the neurovascular unit (NVU, reviewed in [4]), currently available mousee BBB models are largely composed of primary or immortalized BEC lines. Although these models have contributed valuable insights into the cellular and molecular biology of this specialized endothelium, they have limitations as models for BBB drug screening and transport evaluation [7]. Primary mouse BECs have limited scalability and are prone to a rapid loss of BEC phenotype in culture, whereas immortalized mouse BECs (e.g., bEnd.3) are readily scalable but suffer from suboptimal barrier properties in culture such as low baseline TEER values and discontinuous tight junctions [8]. Since the mouse is the most widely used pre-clinical model for discovery and evaluation of brain delivery ‘shuttles’, mouse BBB models in vitro are better surrogates to correlate with mouse studies in vivo than models developed from other species. Furthermore, mouse BBB models may also be more suitable for evaluating BBB changes in neurodegenerative disorders, brain cancers, and inflammation because these diseases are commonly investigated in mouse animal models [9].

In this manuscript, we describe the development and characterization of mouse embryonic stem cell (mESC-D3)-derived BEsC (mBECs) and their application in modeling the BBB in vitro overcoming some of the deficiencies of existing mouse BBB models. Comparative studies of an antibody panel against RMT receptors in mBEC and human iPSC-derived BBB modesls demonstrate the utility of this mouse BBB model in discriminating species-selective antibodies and species-selective transporter properties.

## Materials and methods

### mES culture and BEC differentiation

**Fig. 1:**
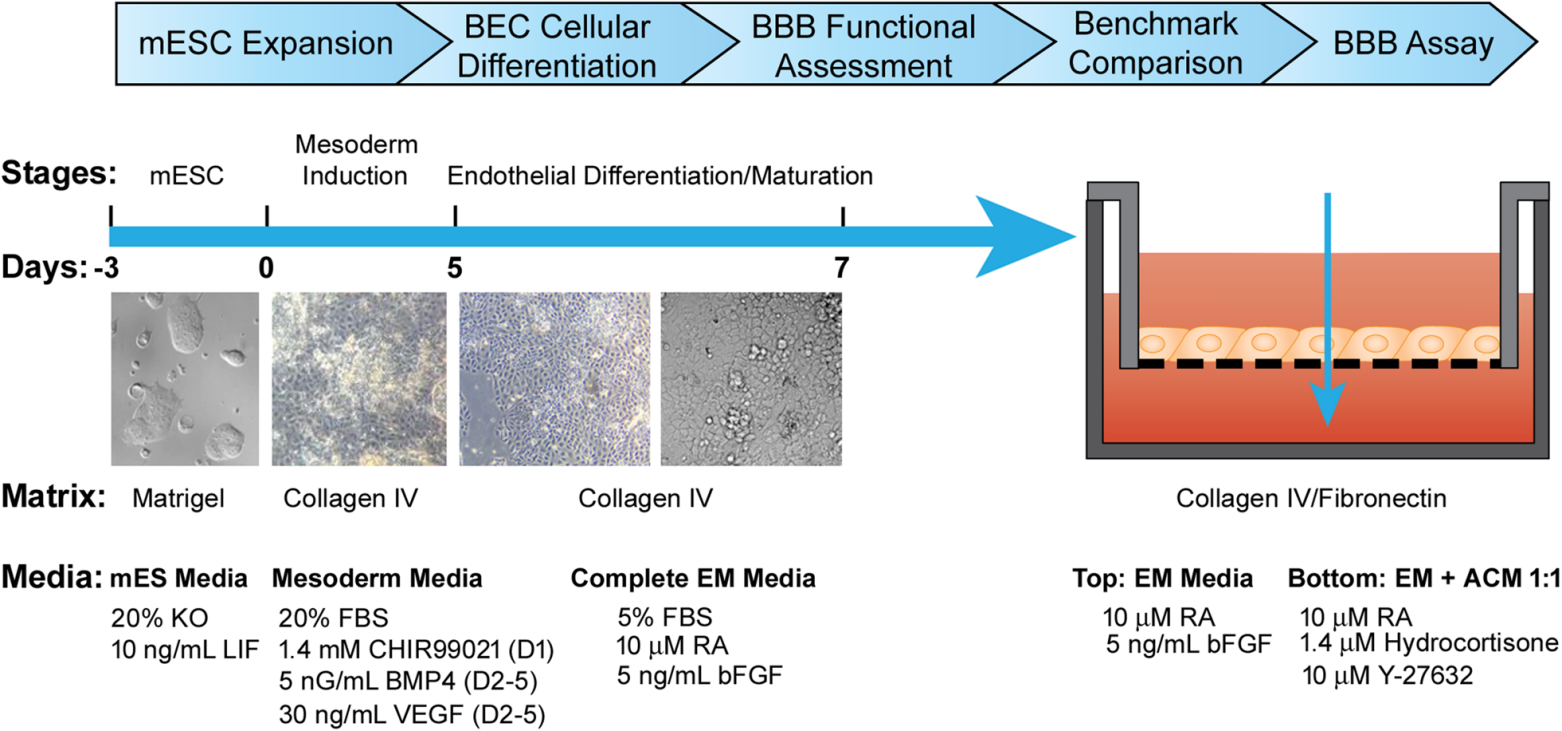
Schematic detailing mESC-D3 differentiation strategy to generate brain-endothelial-like cells and transwell BBB model in vitro. Schematic diagram of mESC-D3 directed monolayer differentiation protocol via initial mesodermal induction and subsequent endothelial cell differentiation and maturation. Representative phase contrast images illustrating morphological changes accompanying the various stages (Days) of differentiation

Mouse embryonic stem cells ESC- D3 (mESC-D3, ATCC, Manassas, Virginia) were adapted to feeder free culture on 0.1% gelatin coated plates in ESGRO-2i medium (Sigma-Aldrich, St. Louis, Missouri). Prior to initiation of mBEC differentiation, the ESC-D3 cells were plated onto Matrigel hESC qualified matrix (Corning, Canton, New York) coated plates in mES medium: DMEM/Ham’s F12 supplemented with Glutamax (Thermo Fisher Scientific), 20% Knock out serum replacement (Thermo Fisher Scientific), 1X MEM-NEAA (Thermo Fisher Scientific, Waltham, Massachusetts), 0.1 mM β-mercaptoethanol (Thermo Fisher Scientific, Waltham, Massachusetts) and 10 ng/ml Recombinant Mouse LIF Protein (R&D Systems, Minneapolis, Minnesota). The ESC-D3 cells were expanded and banked in the mES medium where they maintained their pluripotency markers (Additional file 1: Figure S1). Prior to differentiation, mES were dissociated into a single cell suspension with Accutase (Stem Cell Technologies, Vancouver, British Columbia) and plated at a density of 4 × 10^4^ cells/cm^2^ onto 10 ng/ml Collagen IV (Sigma-Aldrich, St. Louis, Missouri) coated plate in mesoderm induction medium: DMEM/Ham’s F12 supplement with Glutamax (Thermo Fisher Scientific, Waltham, Massachusetts), 20% Fetal Bovine Serum (Hyclone, Logan, Utah), 1X MEM-NEAA (Thermo Fisher Scientific, Waltham, Massachusetts), 0.1 mM b-mercaptoethanol (Thermo Fisher Scientific, Waltham, Massachusetts), 5 ng/ml BMP4 (R&D System), 30 ng/ml VEGF (R&D System, Minneapolis, Minnesota) and 1.4 mM CHIR99021 (Stem Cell Technologies, Vancouver British Columbia). CHIR99021 was only added during the first day of mesoderm induction and after 24 h, the media was replaced with mesoderm media supplemented with 5 ng/ml BMP4 and 30 ng/ml VEGF for an additional 4 days. The mesoderm induction media was changed daily. To induce endothelial differentiation and maturation, the medium was switched to complete Endothelial Media (EM): Mouse Brain Endothelial Cell Culture Serum Free Media (Celprogen, Torrance, California), 5% Fetal Bovine Serum (Hyclone, Logan, Utah), 5 ng/ml bFGF (Thermo Fisher Scientific, Waltham, Massachusetts) and 10 mM *all-trans* Retinoic Acid (RA; Sigma-Aldrich, St. Louis, Missouri) on day 6. After 2 days of culture in EM medium, the cells were dissociated with 0.05% Trypsin–EDTA (Wisent, St-Bruno, QC, Canada) and filtered through a 40 µm sieve to eliminate residual basement membrane and endothelial cell clusters. mBEC were plated at a density of 7.5 × 10^5^ cells/cm^2^ or 1 × 10^6^ cells/cm^2^ onto Collagen IV (80 µg/ml, Sigma-Aldrich, St. Louis, Missouri) and Fibronectin (20 µg/ml; Sigma-Aldrich, St. Louis, Missouri), or Laminin 521(10 µg/ml; Stem Cell Technologies, Vancouver, British Columbia), or Laminin 211(10 µg/ml; Biolamina, Sundbyberg, Sweden) or Laminin 511 (10 µg/ml; AMSBIO, Abingdon, UK) coated Transwell inserts (1.12 cm^2^ cell growth area with 1 µm pore size; Corning, Canton, New York) in complete EM media containing 10 µM RA and 5 ng/ml bFGF. The complete EM media, post-seeding on inserts, was changed daily. Upon seeding of the mBECs onto the Transwell inserts, the inserts were placed into the companion plates containing 1 ml of Astrocyte conditioned medium (see Astrocyte conditioned media section), 1 ml of EM supplemented with 10 µM Y-27632, 1.4 µM Hydrocortisone (Sigma-Aldrich, St. Louis, Missouri), 10 µM RA and 5 ng/ml bFGF to induce barrier formation and tightening. Post plating, mBEC phenotype and function was assessed via immunofluorescence, flow cytometry and TEER measurements. The culture protocol is illustrated in Fig. 1.

### Rat astrocyte conditioned media

Immortalized neonatal rat astrocytes were established in house by SV-40 transfection of primary neonatal rat astrocytes (SV-NRA), isolated from 2 to 4 day old Sprague–Dawley rats. The SV-NRA were grown to 80% confluency in DMEM (Wisent, St-Bruno, QC, Canada) containing 10%FBS (Fisher Scientific, Hampton, NH) and Antibiotic/Antimycotic (Wisent, St-Bruno, QC, Canada). The cells were washed twice with HBSS (Wisent, St-Bruno, QC, Canada) and incubated in 10 ml DMEM and 1% FBS per T75 flask for 72 h. The astrocyte conditioned medium (ACM) was collected, pooled, filter sterilized and aliquoted for storage at −20 ℃.

### Transendothelial electrical resistance (TEER) measurements

Barrier formation of each mBEC-seeded transwell insert was assessed by measuring transendothelial electrical resistance (TEER) prior to being used in the BBB transport assays. A CellZscope apparatus (Nanoanalytics, Potsdam, Germany) was used to conduct the TEER measurement. The values were normalized by subtracting the background (TEER of the empty inserts) and reported in Ω cm^2^, as previously described [10].

### Sodium fluorescein permeability assay

To assess barrier formation of the mBECs, the transwell inserts were washed with 1 ml 1X Hank’s buffered saline solution (HBSS; Wisent, St-Bruno, QC, Canada). The inserts were then placed into companion plates with 1 ml of transport buffer (5 mM MgCl_2_ and 10 mM HEPES in HBSS, pH 7.4) and incubated at 37 ℃ for 10 min and then 500 µl of the transport buffer was removed from the apical chamber of each insert and replaced with 250 µl of sodium fluorescein (50 µg/ml; Sigma-Aldrich, St. Louis, Missouri) in transport buffer. The plates were then incubated at 37 ℃ using the 311DS Labnet (Labnet International Inc.) incubator containing an orbital shaking platform set at 20 rpms for one hour. Sample collection was performed by removing 100 µl of transport buffer from the bottom of the wells at 15, 30, 45 and 60 min intervals for permeability analysis; 100 µl transport buffer was added back to the wells and the plates were returned to the incubator. Inserts without mBEC were used for the background controls. The quantitation of sodium fluorescein was measured using a fluorescent plate reader (excitation 485 nm and excitation 530 nm) and plotted against a standard curve (0–50 ng sodium fluorescein solution in transport buffer), as previously described [10].

### Sucrose permeability assay

An input solution of 1 μCi/ml (0.0025 μCi/ml) of 14C-Sucrose (Perkin Elmer, Waltham, Massachusetts) was prepared in transport buffer (5 mM MgCl_2_ and 10 mM HEPES in HBSS, pH 7.4) and warmed to 37 ℃. The radiolabeled sucrose was dissolved in ethanol, as per manufacturer’s instructions, and three blank inserts were used in each experiment. The 12-well transwell inserts (1.12 cm^2^ cell growth area with 1 µm pore size; Corning, Canton, New York) containing a confluent monolayer of mBECs were dipped sequentially for three consecutive washes of 5–10 min in wells containing 2 ml pre-warmed HBSS to remove any residual medium. The inserts were then placed into companion plates containing 2 ml of pre-warmed transport buffer, equilibrated to 37 ℃ in an incubator for 5–10 min and then 500 µl of the media was carefully removed from the top (apical) chamber of each insert and replaced with 500 µl of the input sucrose solution for a final concentration of 0.00125 µCi/ml. The inserts were incubated at 37 ℃ with gentle rotation using the 311DS Labnet (Labnet International Inc.) incubator containing an orbital shaking platform set at 20 rpms for an hour and 100 µl of transport buffer was collected from the bottom (basal) wells at 15, 30, 45 and 60 min intervals for permeability analysis. Following each collection, 100 µl pre-warmed transport buffer were added back to the bottom wells and the plates were returned to the incubator. The sample collection was also carried out in 3 inserts without cells at 3, 7, 10, 15, 20, 30, 45 and 60 min and clearance slopes were calculated from the linear portion of the curves (0–30 min). Samples were collected in 24-well microbeta sample plates (Perkin Elmer). Duplicate input was also collected by adding 10 µl of the input solution to each well and 90 µl transport buffer. For quantitation, 400 µl high aqueous capacity scintillation fluid was added to each sample well, the plate was covered with a microbeta plate seal and contents were mixed well by gently shaking the plate until solution was homogeneous and clear. The amount of radioactivity per sample was counted in a scintillation counter (Perkin Elmer, Waltham, Massachusetts), using a normalized protocol: 14C channel, 2 min per well, disintegration per minute (dpm). Pe values were calculated as previously described [10].

### Antibody transcytosis assay

A 2X input solution (2.5 µM of each test antibody) (Table 1), was prepared in transport buffer (5 mM MgCl_2_ and 10 mM HEPES in HBSS, pH 7.4) and warmed to 37 ℃. The 12-well transwell inserts (Corning, Canton, New York) containing a confluent monolayer of mBECs were dipped sequentially for three consecutive washes for 5–10 min in wells containing 2 ml pre-warmed HBSS to remove any residual medium. The inserts were then placed into companion plates containing 2 ml of pre-warmed transport buffer, equilibrated to 37 ℃, in an incubator for 5–10 min and 500 µl of the media was carefully removed from the top (apical) chamber of each insert and replaced with 500 µl of 2X input solution (final concentration of 1.25 µM). The inserts were incubated at 37 ℃ with gentle rotation using the 311DS Labnet (Labnet International Inc.) incubator containing orbital shaking platform set at 20 rpms for an hour and 100 µl of transport buffer was collected from the bottom (basal) wells after 90 min for permeability analysis. Following each collection, 100 µl pre-warmed transport buffer were added back to the bottom wells and the plates were returned to the incubator. Where applicable, recombinant holo-transferrin (Tf, Sigma-Aldrich, St. Louis, Missouri) was added in the top (apical) chamber of each insert at a final concentration of 2 mg/ml.

**Table 1:** RMT targeting antibodies used in the mBEC and iBEC BBB transcytosis assays.

Antibody	Type	Receptor	Affinity^a^	Species-cross-reactivity
FC5-Fc	V_H_H-Fc (80 kD)	TMEM30A	50 nM (L-M)	Mouse-rat-human-dog-NHP
J05-Fc	V_H_H-Fc (80 kD)	TfR	300 nM (L)	Mouse-rat-human
8D3	IgG (150 kD)	TfR	1.2 nM (H)	Mouse
8D3v2	IgG (150 kD)	TfR	130 nM (L)	Mouse
IGF1R5-FC	V_H_H-Fc (80 kD)	IGF1R	1 nM (H)	Mouse-rat-human
A20.1	V_H_H (13 kD)	C. Diff Toxin A	2 nM (H)	No mammalian target

^a^*L* Low, *M* Moderate, *H* High Affinity

### Quantification of antibodies in transport assay using multiple reaction monitoring (MRM)

All antibodies and proteins collected from the transport studies, as described above, were reduced, alkylated and trypsin digested using a previously described protocol [11, 12]. For isotopic dilution-based quantification, isotopically heavy versions of the peptides were synthesized from a commercial source (New England Peptide LLC, Gardner, MA) that contained heavy C-terminus K (+ 8 Da). To develop the SRM assay for proteins, each protein was first analyzed by nanoLC-MS/MS using data-dependent acquisition to identify all ionizable peptides. For each peptide, 3 to 5 of the most intense fragment ions were chosen. An initial MRM assay was developed to monitor these fragments at attomole amounts of the digest (about 100–300 amol). Fragments that showed reproducible intensity ratios at low amounts (i.e., had Pearson r2 ≥ 0.95 compared to higher amounts) were considered stable and were chosen for the final MRM assay (Additional file 2: Table S1). The apparent permeability coefficient (P_APP_) values were calculated, as described previously [11, 12].

### Flow cytometry

The mBECs were dissociated with 0.05% Trypsin–EDTA (Wisent, St-Bruno, QC, Canada) and fixed in 4% paraformaldehyde (PFA; Sigma-Aldrich, St. Louis, Missouri) for 15 min at room temperature and washed with 1% bovine serum albumin (BSA; Sigma-Aldrich, St. Louis, Missouri)/ PBS (Wisent, St-Bruno, QC, Canada). The cells were subsequently incubated with cold 10% methanol (Sigma-Aldrich, St. Louis, Missouri) at 4 ℃ for 20 min and then washed with 1% BSA/PBS. The cells were blocked with CD16/CD32 Monoclonal Antibody (1:100; Thermo Fisher Scientific, Waltham, Massachusetts) for 10 min and incubated with fluorescently conjugated antibodies for 30 min at room temperature and then washed with 1% BSA/PBS. Fluorescence was acquired with the BD LSRFortessa flow cytometer (BD Biosciences, Franklin Lakes, New Jersey). Forward- and side-scatter on unstained control were used to gate cells, respectively. Forward-scatter height vs. forward-scatter area was used to gate on single cells. Analysis was performed using FlowJo software. Details on antibody source and dilution are provided in Additional file 2: Table S2.

### Endothelial angiogenesis assay

The mBEC were dissociated with 0.05% Trypsin–EDTA (Wisent, St-Bruno, QC, Canada) and plated at a concentration of 2.5 × 10^5^ cells in EM containing 50 ng/mL VEGF (R&D System, Minneapolis, Minnesota) in a 24-well tissue culture plate coated with 250 µl of Matrigel Basement Membrane Matrix (Corning, Canton, New York). The endothelial cell tube formation was visualized by CFDA staining (2.5 µg/ml) in Live Cell Imaging Buffer (Thermo Fisher Scientific, Waltham, Massachusetts) for 30 min at 37 ℃. The cells were washed with cold PBS and imaged using a 20X HMC objective on an Axiovert 300 M Microscope (Zeiss).

### Immunocytochemistry

BEC cells were grown in 12 well plates on 15 mm round coverslips coated with either rat-tail Collagen or Collagen IV & Fibronectin in the respective growth media. For most antigens, the cells were fixed using Genofix (DNA Genotek, Ottawa, Ontario). In the latter case, the cells were permeabilized with 0.2% Triton X-100 (Sigma-Aldrich, St. Louis, Missouri) in PBS (without Ca2^+^/Mg2^+^) for 20 min, washed and blocked using DAKO Protein Block Serum Free (Agilent) for 20 min at room temperature. Primary antibodies were prepared using the DAKO Antibody Diluent (Agilent, Santa Clara, California), according to the dilutions described in Additional file 2: Table S2 and coverslips were incubated for 1 h at room temperature in a humidified chamber. Coverslips were then washed three times for a minimum of 5 min with PBS (without Ca2^+^/Mg2^+^) and incubated with secondary antibodies diluted 1:500 in antibody diluent at room temperature for 1 h in the dark. Secondary PBS-only controls were performed in parallel for all staining experiments. The coverslips were then washed three times for 5 min with PBS and mounted using DAKO fluorescent mounting medium (Agilent, Santa Clara, California) spiked with 5 µg/ml of Hoechst 33258 (Sigma-Aldrich, St. Louis, Missouri) to counterstain nuclei. Images were captured using the Axiovert 200 M Microscope (Zeiss). Cells on coverslips were imaged using a 20 x /0.5 Plan Neofluar objective and live cells were imaged using 20 × 0.4 LD Achroplan Korr (DICII) objective.

### RNASeq Analysis

Total RNA was extracted from cell pellets using NucleoSpin RNA plus kit (Macherey–Nagel GmbH & Co. KG) according to manufacturer’s instructions. Genomic DNA contamination was removed by Turbo DNA-Free Kit (Thermo Fisher Scientific, Waltham, Massachusetts). RNA quality was assessed using Agilent Bioanalyzer 2100. RNASeq Libraries were generated using the TruSeq strand RNA kit (Illumina, San Diego, California). The libraries were quantified by Qbit and qPCR according to the Illumina Sequencing Library qPCR Quantification Guide and the quality of the libraries was evaluated on Agilent Bioanalyzer 2100 using the Agilent DNA-1000 chip, as previously described [13]. The RNASeq library sequencing was performed using Illumina Next-Seq500. RNA-seq data in FASTQ file format was processed by trimming the adaptor sequences, filtering low-quality reads (Phred Score <  = 20) and eliminating short reads (length <  = 20 bps) using software package FASTX-toolkit [http://hannonlab.cshl.edu/fastx_toolkit/]. STAR (v2.7.8a) [13] was used for the alignment of reads to the reference genome and to generate gene-level read counts. Mouse (Mus musculus) reference genome (version GRCm39 Gencode M26) [14] and corresponding annotations were used as references for RNA-seq data alignment process (https://www.gencodegenes.org/mouse/stats.html). DESeq2 [15] was used for data normalization. The expression value of each gene was expressed as average read count of three replicates. Heat maps were generated with Graph Pad Prism. Data values were log_2_ transformed.

### Rhodamine123 efflux studies

To assess functional polarization of transporter activity in mBECs, a substrate of the efflux transporter P-gp, Rhodamine 123 (Sigma-Aldrich, St. Louis, Missouri) was used. The mBEC transwell inserts were placed into plates with 2 ml of transport buffer (5 mM MgCl_2_ and 10 mM HEPES in HBSS, pH 7.4) and incubated at 37 ℃ for 10 min and then 500 µl of the transport buffer was removed from the luminal chamber of each insert and replaced with 500 µl of Rhodamine 123 (20 µM) in transport buffer. The inserts were incubated at 37 ℃ with gentle rotation using the 311DS Labnet (Labnet International Inc.) incubator containing orbital shaking platform set at 20 rpms for an hour, and 100 µl of transport buffer was collected from the luminal chamber of each insert and bottom of the wells at 15, 30, 45 and 60 min intervals for permeability analysis, 100 µl transport buffer were added back to the inserts and wells and the plates were returned to the incubator. Inserts without mBEC were used for the background controls. The quantitation of Rhodamine 123 was performed using a fluorescent plate reader (ex., 508 nm and em., 528 nm) and plotted against a standard curve (0–10 µM Rhodamine 123 in transport buffer).

### Human HAF-iPSC-BBB (iBEC) model

Amniotic fluid cell (HAF) derived iPSC (HAF-iPSC) were differentiated into brain endothelial like cells (iBECs), as previously described [10, 16]. In brief, during the initial pre-differentiation step, once the HAF-iPSCs reached 60–70% confluency the medium was switched from mTeSR1 to low osmolality KOEB medium composed of KnockOut DMEM/F12 medium (Thermo Fisher Scientific) supplemented with 20% Knock-Out serum replacement, 1 X Glutamax, 1X non-essential amino acids and 55 µM β-mercaptoethanol (all from Thermo Fisher Scientific, Waltham, Massachusetts) for 5–7 days. During this time frame, major morphological changes were observed as the cells became bigger and began to assume a cobblestone-like morphology. Once the cells formed a uniform monolayer of endothelial-like cells, the medium was switched to endothelial differentiation medium (EM) composed of human serum free endothelial medium (Thermo Fisher Scientific, Waltham, Massachusetts) supplemented with 1% Fetal bovine serum (Hyclone, Logan, Utah) and 20 ng/ml bFGF for 9–10 days. While in EM culture, the cells acquired a typical cobblestone morphology characteristic of differentiated endothelial cells. After 9–10 days in EM, the cells were dissociated with Accutase (Stem Cell Technologies, Vancouver, British Columbia) at 37 ℃ for 10–15 min. Once dissociated, the cells were filtered through a 40 µm sieve to eliminate residual basement membrane and endothelial cell clusters and re-suspended in EM containing 10 µM ROCK Inhibitor. Singularized iBECs were plated onto 0.5% gelatin coated transwell inserts as described under the “Preparation of transwell inserts and TEER measurements” section. All-trans 10 µM RA was added following seeding of iBECs onto inserts where indicated. A mixture of collagen IV (80 µg/ml; Sigma-Aldrich, St. Louis, Missouri) and fibronectin (20 µg/ml; Sigma-Aldrich, St. Louis, Missouri) coated on transwell inserts was used where indicated. All iBEC characterizations were performed at the end of the differentiation process (21 days from iPSCs) following passage onto gelatin coated inserts and coverslips, where appropriate.

### SV-ARBEC BBB model

Immortalized adult rat brain microvascular endothelial cells, SV-ARBECs, were established by SV-40 transfection of primary rat brain microvascular endothelial cells, isolated from 24 to 30 day old Sprague–Dawley rats, as previously described [17]. The cells were grown in M199 medium (Wisent, St-Bruno, QC, Canada) supplemented with 10% FBS and antibiotic/antimycotic and routinely passaged at a split ratio of 1:20 every week. For transport studies, the SV-ARBEC cells were seeded at 80,000 cells on rat-tail collagen I (VWR, Mississauga, ON)-coated 1.12 cm^2^ 1 µm pore size inserts, in 1 ml SV-ARBEC maintenance medium (Thermo Fisher Scientific, Waltham, Massachusetts). The bottom of the companion plate contained 2 ml of 1:1 ratio of Maintenance Medium and Rat Astrocyte Conditioned Medium produced in-house. The SV-ARBEC BBB model characterization and transport experiments were performed, as previously described [12, 17].

### bEnd.3 BBB model

bEnd.3 cells were purchased from ATCC (Manassas, VA) and cultured in Maintenance Medium containing DMEM (Wisent, Saint-Jean-Baptiste, QC) supplemented with10% FBS (Fisher Scientific, Hampton, NH) and Antibiotic/Antimycotic (Wisent, St-Bruno, QC, Canada). The cells were passaged weekly at 1:3 ratio. For the transport studies, the bEnd.3 cells were seeded at 80,000 cells on collagen I (VWR, Mississauga, ON)-coated 1.12 cm^2^ Falcon cell inserts, 1 µm pore size, in 1 ml maintenance medium. The bottom of the companion plate contained 2 ml of 1:1 ratio of Maintenance Medium and Rat Astrocyte Conditioned Medium produced in-house. The cells were allowed to grow to confluence for 17 days with full media change in the insert every 4 days and the bottom chamber every 7 days.

### Primary mouse BBB model

Mouse Primary Brain Microvascular Endothelial Cells (mPBMEC) were purchased from Cell Biologics (Chicago, IL) and were propagated using Endothelial Cell Complete Medium (Cell Biologics, Chicago, IL) on 0.5% gelatin (Sigma Aldrich, St. Louis, MO) coated flasks (VWR, Randor, PA). For the transport studies, the mPBMECs were seeded at 80,000 cells collagen I (VWR, Mississauga, ON)-coated 1.12 cm^2^ Falcon cell inserts, 1 µm pore size, in 1 ml SV-ARBEC feeding medium. The bottom of the companion plate contained 2 ml of 1:1 ratio of Endothelial Cell Complete Medium (Cell Biologics) and Rat Astrocyte Conditioned Medium produced in-house. The mPBMEC were allowed to grow to confluence on the inserts for 5 days.

### Wes western blot

Cell pellets were lysed in RIPA buffer (Sigma-Aldrich, St. Louis, Missouri) both containing 1 X Complete protease inhibitor (Roche, Basel, Switzerland). After 30 min incubation on ice, lysates were vortexed then centrifuged at 21 000 × *g* for 10 min in a Sorvall Legend Micro 21R centrifuge. Protein concentrations were determined using the Quantipro BCA Assay Kit (Sigma-Aldrich, St. Louis, Missouri). Wes was run using the 12–230 kDA separation module and the mouse or rabbit detection module (ProteinSimple). Wes samples (0.8 mg/ml) were prepared by combining Master Mix to sample in a 1:4 ratio. Samples and Biotinylated Ladder were heated in an Accublock digital dry bath at 95 ℃ for 5 min. Samples were cooled to room temperature, vortexed to mix and centrifuged in a Mandel mini microfuge. Biotinylated ladder, samples, primary and secondary antibodies, and luminol were loaded on the plate and Wes was run, as previously described [18]. Primary antibodies were mouse anti-transferrin receptor (Thermo Fisher, 13–6800, 1:10; Thermo Fisher Scientific, Waltham, Massachusetts), rabbit anti-LRP1 (Abcam, ab92544, 1:200; Waltham, Massachusetts), rabbit anti-TMEM30A (Abcam, Ab105062, 1:10; Waltham, Massachusetts), rabbit anti-insulin receptor (Cell Signalling, 3025 T, 1:100; Danvers, Massachusetts), and anti-actin-HRP (Sigma, A3854, 1:200; Sigma-Aldrich, St. Louis, Missouri). Streptavidin-HRP was used to detect the ladder proteins.

### mBEC activation

mBECs were seeded at density of 7.5 × 10^5^ cells/cm^2^ on a 24 well plate that were pre-coated with Collagen IV (80 µg/ml; Sigma-Aldrich, St. Louis, Missouri) and Fibronectin (20 µg/ml; Sigma-Aldrich, St. Louis, Missouri) in complete EM medium with 10 µM Y27362 (ROCK Inhibitor; Stem Cell Technologies, Vancouver, British Columbia). mBECs were stimulated with 300 ng/ml of recombinant human TNF-α (R&D Systems, Minneapolis, Minnesota) and 200 IU/ml recombinant human IFN-γ (R&D Systems, Minneapolis, Minnesota) for 24 h. Activated and non-activated control cells were dissociated with 0.05% Trypsin–EDTA (Wisent, St-Bruno, QC, Canada) and washed with 1% bovine serum albumin (BSA; Sigma-Aldrich, St. Louis, Missouri)/ PBS (Wisent, St-Bruno, QC, Canada). Cells were blocked with anti CD16/CD32 monoclonal antibody (1:100; Thermo Fisher Scientific, Waltham, Massachusetts) for 10 min and stained with fluorescently-conjugated antibodies (Additional file 1: Figure S2) for 30 min at room temperature and then washed with 1% BSA/PBS. Cells were acquired with the BD Accuri C6 Plus flow cytometer (BD Biosciences). Forward- and side-scatter on unstained control were used to gate on cells, respectively. Forward-scatter height vs. forward-scatter area was used to gate on single cells. Analysis was performed using FlowJo software.

### Statistical analysis

At least three independent differentiations and three technical experiments were performed unless otherwise specified in the figure legends. Results are given as mean ± standard deviation (SD). Statistical test are indicated in Figure legends and level significance was set at p < 0.05, indicated with asterisk (*). Grading in significance is indicated as follows: *p < 0.05, **p < 0.01, ***p < 0.001.

## Results

### Differentiation of mouse ESC-D3 cells to brain endothelial cells (mBECs)

We developed a two-step directed monolayer differentiation strategy to differentiate mouse ESC-D3 (mESC-D3) cells into mouse brain endothelial-like cells (mBECs) (Fig. 1). mESC-D3 cells were adapted to and cultured in feeder-free conditions maintained on Matrigel coated plates in chemically-defined serum-free mES medium containing DMEM/Ham’s F12 media with 20% Knock-Out serum replacement supplemented with 10 ng/ml Recombinant Mouse LIF protein [19]. Upon transitioning to Matrigel, the mESC-D3 colonies retained high proliferation rates, dense and flattened colony formation and high expression of pluripotency markers Oct4, Sox2 and Nanog (Additional file 1: Figure S1).

**Fig. 2:**
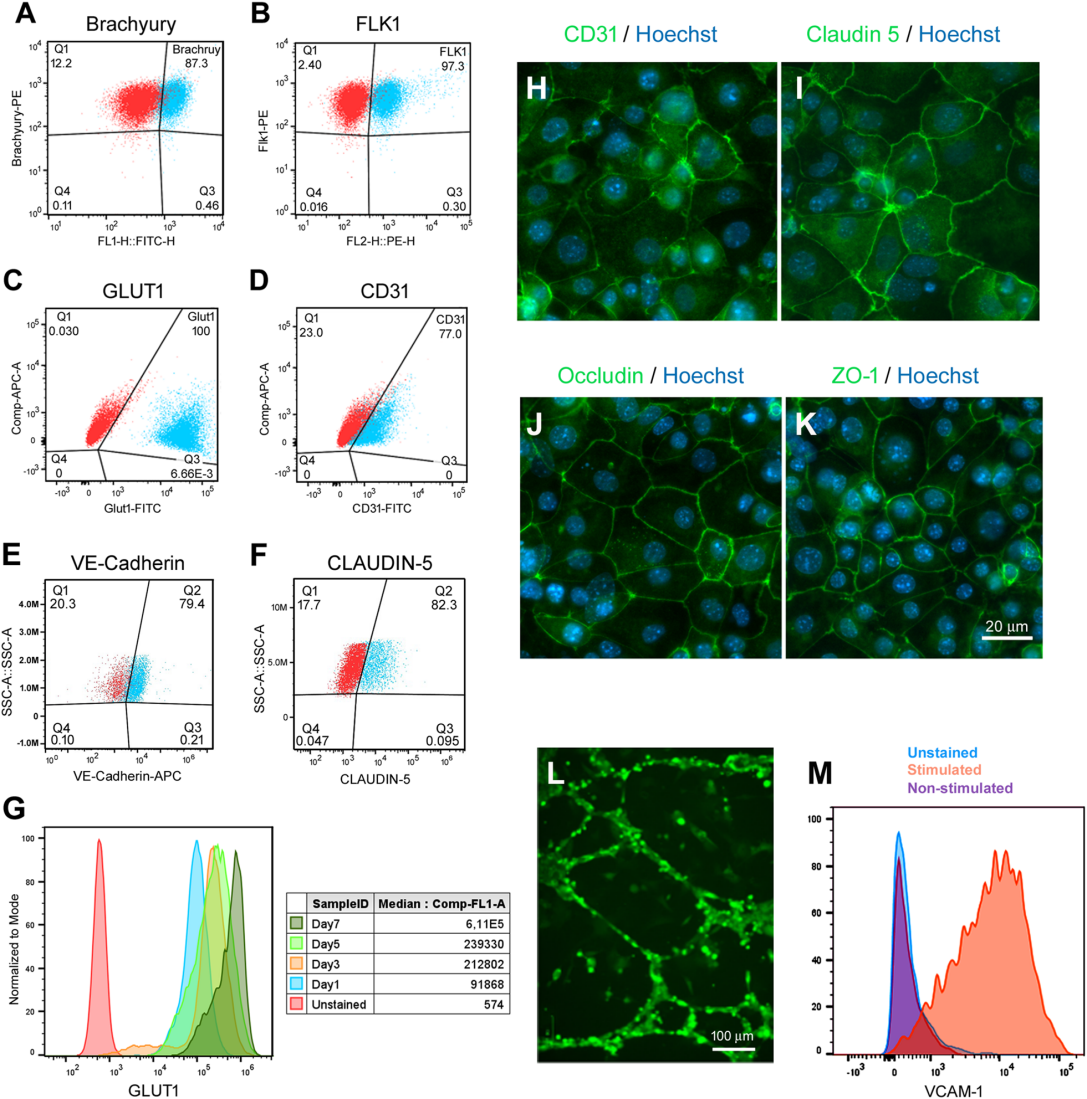
Differentiation of mESC-D3 into mouse brain endothelial like-cells (mBECs). Flow cytometry analysis assessing transitional differentiation stages of endothelial differentiation. **A** Brachyury expression was assessed after 24 h of mesodermal induction with 5 ng/ml BMP4 and 30 ng/ml VEGF and **B** endothelial progenitor Flk1 expression was assessed after 5 days in endothelial differentiation media. **C**–**F** GLUT1, CD31, VE-Cadherin and CLAUDIN 5 expression was assessed at the end of the differentiation period (Day 8). Red = unstained controls. **G** Temporal GLUT1 expression was assessed during the entire differentiation period. Increasing GLUT1 expression is shown with MFI (inset) at Day 1, 3, 5 and 7. The terminally differentiated mBECs exhibit cobblestone monolayer morphology and stained positive for key **H** endothelial (CD31, green) and (**I**–**K**) BBB-specific tight junction proteins (Claudin 5, Occludin and ZO-1; all green). Hoechst counterstain (blue); Scale bar = 20 µm. **L** In the presence of VEGF, the mBEC formed vascular–like structures in Matrigel assay within 24 h. Green = CFDA staining; Scale bar = 100 µm. **M** Following stimulation with inflammatory cytokines, the mBECs expressed immune adhesion molecule VCAM-1. Blue = unstained, Purple = non-stimulated; Orange = TNF-α  and INF-γ stimulated. Representative images shown of 5 independent differentiations

Differentiation of mESC towards endothelial cell types has been shown to be driven by VEGF, BMP4 and bFGF. At early stages of commitment, BMP-4 and VEGF are known to promote ventral mesoderm and endothelial specification while inhibiting neuronal development [20–22]. At low levels, BMP-4 induces mesoderm and subsequent endothelial cell differentiation from Flk1^+^ cells [22]; whereas bFGF is more important in later stages of endothelial differentiation for enhancing endothelial cell proliferation [21, 23, 24]. Extracellular matrix (ECM) substrates have also been shown to play a role in endothelial cell induction with collagen-type IV showing improved vascular endothelial cell differentiation of mouse ESCs [23, 25]. To induce mesoderm differentiation, mESC-D3 cells were dissociated into a single cell suspension and re-plated at a density of 4 × 10^4^ cells/cm^2^ onto Collagen type-IV coated plates in mesoderm induction media containing DMEM, 20% FBS, 30 ng/ml of VEGF and 5 ng/ml BMP4. Since activation of Wnt signaling has been shown to direct human iPSCs towards mesodermal endothelial progenitors [26, 27] and promote the acquisition of BBB-specific properties in vivo [28–30], we supplemented the mesoderm induction media with 1.4 mM canonical Wnt agonist CHIR99021 during the first day of mesoderm induction, as previously described [26]. After 24 h of CHIR99021 treatment, the cells expressed very high levels of mesoderm marker Brachyury (87.3%, Fig. 2a, Additional file 1: Figure S2). On day 2, CHIR99021 was removed and the cells were maintained for an additional 3 days in mesoderm induction media in the presence of 5 ng/ml BMP4 and 30 ng/ml VEGF. By day 5, the majority of the cells expressed endothelial progenitor marker Flk1 (97.3%, Fig. 2b). Given this high fidelity of endothelial progenitor differentiation, no enrichment of Flk1 positive cells was required before transitioning to endothelial differentiation and maturation. At day 6, the medium was changed to complete endothelial medium (EM) composed of serum-free mouse brain endothelial cell culture media (Celprogren) supplemented with 5% FBS, 5 ng/ml bFGF and 10 µM retinoic acid (RA). Since activation of RA signaling pathways has been shown to play a critical role in endothelial progenitor specification to BECs and acquisition of BBB properties [31, 32], we supplemented the EM with 10 µM RA during endothelial differentiation (Fig. 1). Following 2 days in complete EM culture, the cells showed a very homogenous and cobblestone-like morphology. At day 8, the cells were dissociated and flow cytometry analysis confirmed robust expression of endothelial and BBB markers such as glucose transporter 1 (GLUT1; 100%, Fig. 2c), PECAM-1 (CD31; 77%, Fig. 2d), VE-Cadherin (CDH5; 79.4%, Fig. 2e) and CLAUDIN 5 (82.3%, Fig. 2f). To monitor the differentiation process, we examined the temporal expression of brain-type GLUT1 by flow cytometry and observed an increase in GLUT1 expression during the consecutive stages of BEC specification and maturation (Fig. 2g), as previously described [10, 33]. Expression of CD31 and tight junction proteins Claudin 5, Occludin, and Zonula Occludens (ZO-1) was also confirmed by immunofluorescence (Fig. 2h–k, Additional file 1: Figure S3a). Henceforth, we refered to these cells as mESC-D3 derived brain endothelial cells (mBECs).

We subsequently compared the mBEC expression profile for CD31, Claudin 5, Occludin and ZO-1 with immortalized mouse brain endothelial cells (bEnd.3) and primary mouse brain endothelial cells (pmBEC) using immunofluorescence staining (Additional file 1: Figure S3b). By contrast to mBECs (Additional file 1: Figure S3a), bEnd.3 and pmBEC showed more discontinuous tight junction marker expression (Additional file 1: Figure S3b). Of note, the Claudin 5 antibody (Clone 4C3C2) used in these studies has been validated for Claudin 5 specificity, as previously described [34, 35] (see also Additional file 1: Figure S3C). These observations support the described loss of BBB-specific phenotypes of these BEC lines in culture [8]. The vascular phenotype of the mBECs was further confirmed by formation of vascular tube-like structures in the presence of VEGF (Fig. 2l) and VCAM-1 upregulation in response to inflammatory stimulus (Fig. 2m).

### Functional barrier formation

To assess functional barrier formation, the mBECs were evaluated in a two-compartment in vitro Transwell BBB assay. Following 2 days in EM culture, the mBECs were dissociated and seeded at a density of 7.5 × 10^5^ cells/cm^2^ onto Fibronectin/Collagen IV coated transwell polyethylene terephthalate (PET) permeable inserts (1 µm pore size) in EM medium containing 5% FBS, 5 ng/ml bFGF and 10 µM RA. Fibronectin/Collagen IV-based matrix selection has been shown to selectively purify endothelial cells during human iPSC-BEC differentiation and promote barrier formation [33]. To induce BBB-specific properties of the mBECs, the inserts were placed in companion plates containing a 1:1 mix of EM media with rat astrocyte conditioned medium (ACM) supplemented with 10 µM Y-27632, 10 µM RA and 1.4 µM Hydrocortisone, which are known to induce barrier tightness in other BBB models (Fig. 1) [10, 37–39]. Barrier formation was assessed, by measuring transendothelial electrical resistance (TEER), as a quantitative measurement indicative of barrier impermeability or “tightness” to paracellular diffusion.

**Fig. 3:**
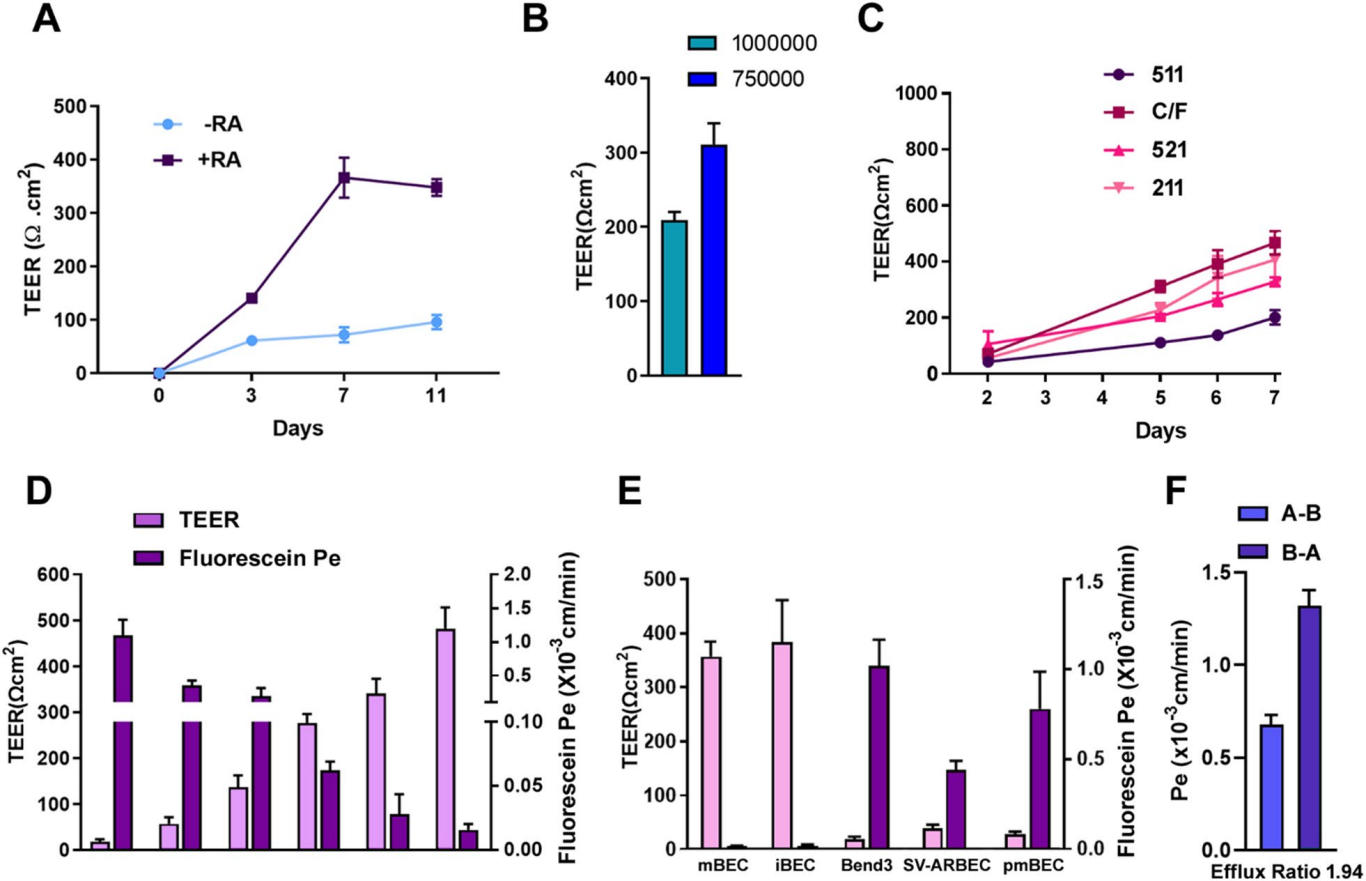
Functional characterization of BBB properties of mBECs. **A** Transendothelial electrical resistance (TEER, Ω cm^2^) of confluent mBEC monolayers on Collagen IV/Fibronectin coated 1 µm pore transwell inserts cultured in the presence or absence of 10 µM *all-trans* Retinoic Acid (± RA) and ACM in the abluminal chamber (mean ± SD) assessed over 11 days in culture. Results are representative of 3 independent differentiations. **B** Comparison of effect of transwell seeding densities on TEER values (mean ± SD). **C** Comparison of laminin matrix coatings: Laminin 511 (511), Laminin 521 (521), Laminin 211 (211), and Collagen IV/Fibronectin (C/F) on TEER induction (mean ± SD) over a 7 day period. Results are representative of 3 independent differentiations. **D** Comparison between TEER values (left y-axis) and sodium fluorescein permeability coefficient (Pe, right y-axis) in mBECs from 6 independent differentiations during protocol optimization (x-axis). The means reflect variability from different differentiations during protocol optimization steps (mean ± SD). **E** Comparison between TEER values (left y-axis) and sodium fluorescein permeability coefficient (Pe, right y-axis) in mBECs, human iPSC-derived BECs (iBECs), bEnd.3, SV-ARBECs and pmBECs (mean ± SD). Results are representative of 6 independent differentiations. **F** Permeability values of Rhodamine123 from apical to basolateral (A-B) and basolateral to apical (B-A) compartments. Efflux ratio (B-A/A-B) for Rhodamine is 1.94 (mean ± SD). Results are from 2 independent differentiations

We assessed the TEER values of mBECs differentiated in the presence or absence of RA. Supplementation of RA during endothelial differentiation resulted in a steady increase in TEER values compared to non-RA treated mBECs (Fig. 3a). Average TEER values for RA-treated mBECs were 141 Ω cm^2^ after 72 h post plating and increased over time to a maximum 350–400 Ω cm^2^ by day 7; thereafter, the TEER values plateaued at 350 Ω cm^2^ by day 11 (Fig. 3a). For the non-RA treated mBECs, TEER values increased only modestly reaching values of 60–100 Ω cm^2^ during the 11 day period (Fig. 3a). RA supplementation during endothelial differentiation also increased the expression of GLUT1, CD31, ZO1, Occludin, Claudin 5 and CDH5 (Additional file 1: Figure S4), collectively contributing to the increase in TEER. These observations are consistent with previous reports showing that RA induces BBB-specific genes and properties in mouse BECs [40]. Similarly, addition of RA during human iPSC-derived BEC specification stage substantially increased both the differentiation efficiency and barrier properties [31]. Since seeding density has also been shown to affect TEER values in human iPSC-BECs [41], we subsequently tested two seeding densities, 7.5 × 10^5^ cells/cm^2^ and 1 × 10^6^ cells/cm^2^ and observed that the latter gives the highest TEER values (Fig. 3b).

### ECM coating and barrier formation

Laminins are known to be a major constituent of the gliovascular basal lamina known to regulate the maturation and function of BBB. Laminin 511 and laminin 211 are secreted by BEC and astrocytes; respectively, at the BBB [42–44]. Laminin 521 and 211 have also been shown to support endothelial cell differentiation and the latter to induce higher TEER properties in iPSC-derived BBB models [47, 46]. We subsequently tested whether Laminin 521, 211 and 511 would improve barrier formation compared to Collagen IV/Fibronectin coated inserts. We found that Laminin 211 (407 Ω cm^2^) induced higher TEER values compared to either Laminin 521 (329 Ω cm^2^) or 511 (201 Ω cm^2^); however, the highest TEER values were observed for Collagen IV/Fibronectin (467 Ω cm^2^) after 8 days on inserts (Fig. 3b, d). These TEER values are similar to those obtained in our HAF-iPSC-BBB (iBEC) model (Fig. 3e) [10]. By comparison, the TEER values for bEnd.3, SV-ARBEC and pmBEC cells were approximately 20, 40 and 30 Ω cm^2^; respectively (Fig. 3e). Commonly reported TEER values for mouse primary BECs ranged from 100–300 Ω cm^2^ [8]; however, these primary lines are known to gradually loose barrier integrity over a couple of passages. Based on these findings, all subsequent analyses were performed using Collagen IV/Fibronectin coated inserts between day 5 and day 10 with TEER value ranging between 300 to 600 Ω cm^2^ (Fig. 3d-e).

### Sodium fluorescein permeability and functional efflux transporters

Sodium fluorescein (NaFl), a low molecular weight marker of paracellular permeability, is routinely used as a benchmark for passive transport in BBB models in vitro. NaFl paracellular permeability (Pe) in mBECs was observed to inversely correlate with TEER values (Fig. 3d). NaFl permeability for the mBECs (Pe = 1.7 × 10^–5^ cm/min) was similar to that observed in our iBEC model (Pe = 2.0 × 10^–5^ cm/min) and substantially lower than that in bEnd.3 (Pe = 1.02 × 10^–3^ cm/min) and SV-ARBEC models (Pe = 0.44 × 10^–3^ cm/min) (Fig. 3e). Similar Pe trends were also observed for sucrose permeability (Additional file 1: Figure S5). Lastly, functional polarization of transporter activity in the mBECs was assessed using Rhodamine 123 as a substrate for the efflux transporter P-gp. The Pe values indicated polarized transport of Rhodamine 123 with A-B Pe = 0.68 ± 0.04 × 10^−3^ cm/min and B-A Pe = 1.31 ± 0.06 × 10^−3^ cm/min with B-A/A-B efflux ratio of 1.94 (Fig. 3f). However, since Rhodamine 123 is also a substrate of OATP1A2 and OCT1 and the efflux ratio of 1.94 is below the threshold of what is considered a P-gp substrate by the FDA (ER > 2), further investigation into the efflux activity of this model would be required.

### Transcriptomic RNASeq profiling of mBECs

**Fig. 4:**
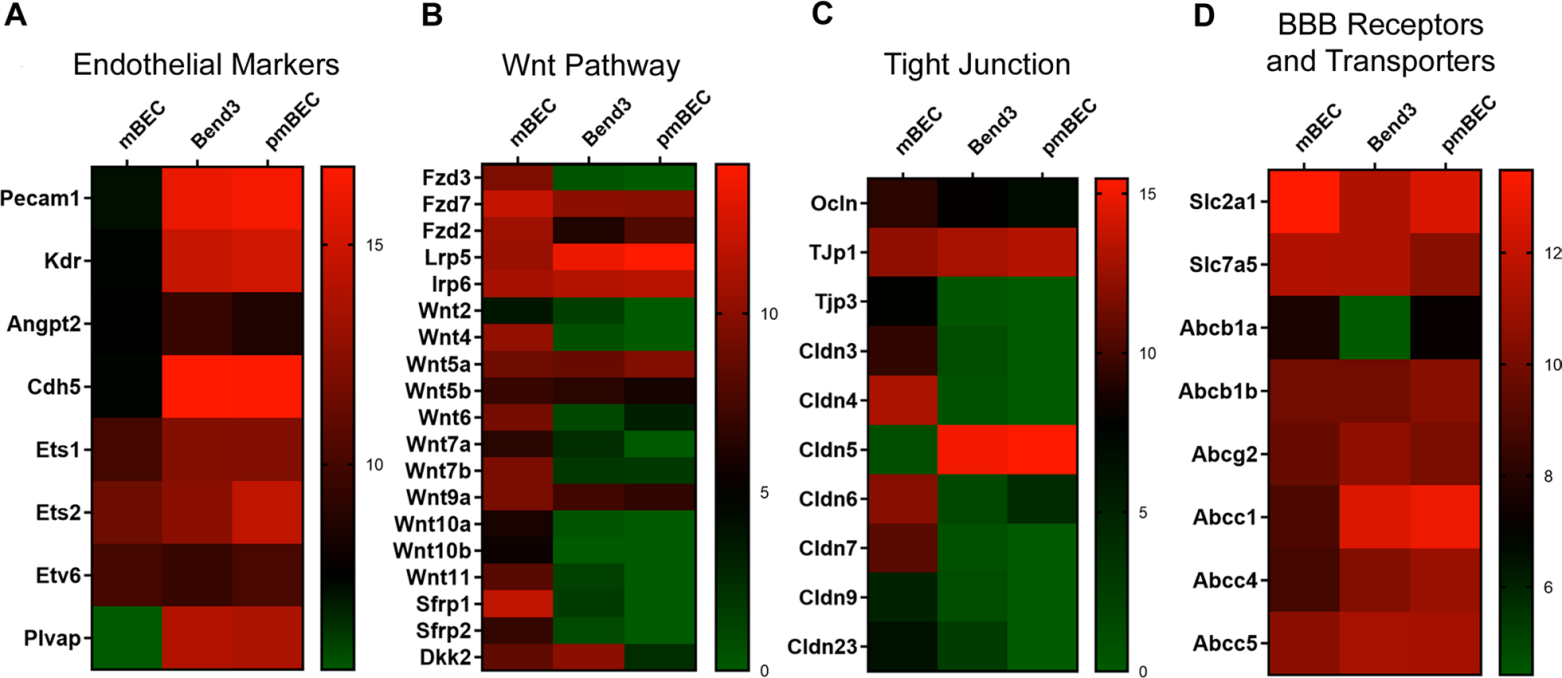
Comparative RNASeq analysis of genes expression profiles in mBEC, bEnd.3 and pmBECs. Heatmaps depicting log2 transformed transcript abundances of **A** endothelial, **B** Wnt signaling, **C** tight junction **D** BBB receptor/transporters gene expression profiles in mBEC, immortalized bEnd.3 and primary mouse brain endothelial cells (pmBECs). Green = low expression; Red = high expression. Results are from three independent differentiations

We subsequently performed RNASeq analysis on the mBEC, bEnd.3 and pmBECs to assess the similarities and differences in their transcriptomic signatures. Similar to pmBEC and bEnd.3, mBECs expressed canonical endothelial cell markers including *Pecam-1* (*CD31*), *Vegfr2* (Flk1/*Kdr*), *Angiopoietin 2* (*Angpt2)* and *VE*-*cadherin* (*Cdh5*)*.* The mBECs also express some critical ETS transcription factors, such as *Ets1, Ets2* and *Etv6,* which are critical for establishing a vascular endothelial identity [47, 48] (Fig. 4a, Additional file 1: Figure S6). Overall, expression levels for these key canonical endothelial markers were lower in the mBECs, as has also been described for human iPSC-derived BECs [48]. This lower expression of canonical endothelial markers has also been described for other iPSC/ESC-derived endothelial cells attributed to a lack of complete functional maturation in vitro [48, 49]. Furthermore, we also observed low expression of endothelial cell specific *Plasmalemma vesicle-associated protein* (*Plvap),* a protein associated with highly permeable blood vessels [50]*.* PLVAP expression in BECs only occurs in pathological conditions associated with a compromised barrier function and increased vesicular transport activity [51]. Similar to the human iPSC-derived BECs [48, 52, 53], the mBECs also express a number of epithelial-associated transcripts reminiscent of the choroid plexus (Additional file 1: Figure S7) highlighting that the differentiation process yields a more epithelial-like phenotype.

Wnt/β-catenin signaling has been identified as a key regulator of CNS angiogenesis, BBB formation and maintenance inducing tight junction, solute transporter and efflux transporter expression [28, 54, 55]. We found that mBECs expressed not only Wnt receptors and co-receptors (*Fzd3, Fzd7, Fzd2, Lrp5 and Lrp6*) but also several Wnt ligands (*Wnt2, Wnt4, Wnt5a, Wnt5b, Wnt6, Wnt7a, Wnt7b, Wnt9a, Wnt10a, Wnt10b, Wnt11*) and Wnt modulators (*Sfrp1, Sfrp2 and Ddk2*) that are involved in barriergenesis (Fig. 4b). Expression of *Wnt2, Wnt5a* and *Wnt11* have been shown to be involved in endothelial commitment of ESCs [56–58] and *Wnt7a* and *Wnt7b* to help promote BBB specification of endothelial cells [30, 55]. These were upregulated in the mBECs compared to bEnd.3 and pmBECs. The mBECs also expressed high levels of *Sox17* (Fig. 4b, Additional file 1: Figure S6), a major downstream transcriptional target of Wnt-β-Catenin, and one of the major transcriptional targets of Wnt/β-catenin during vascular development of the brain microvasculature and BBB integrity [59]. Up-regulation of canonical and non-canonical Wnt and Frizzled genes in Flk1 cells may play an important role in mESC endothelial differentiation [58] and provide novel insight into the molecular basis of endothelial cell differentiation.

We also observed increased expression of tight junction transcripts such as *Occludin* and *Zonula Occludens* (*Zo-1, Zo-3*) (Fig. 4c, Additional file 1: Figure S3 and S6). Transcript levels of *Claudin 5* (*Cldn5*) were lower in the mBECs compared to pmBECs and bEnd.3 cells (Fig. 4c, Additional file 1: Figure S6); however, flow cytometry and immunofluorescence analysis confirmed high expression levels of Cldn 5 (Fig. 2f, Additional file 1: Figure S4) with discrete membrane expression (Fig. 2h) compared to discontinuous expression observed in the pmBEC and bEnd.3 cells (Additional file 1: Figure S3). These observations highlight the importance of validating cell structure and function in addition to global transcriptomic profiling results. Interestingly, we also observed increased expression of a panel of tight junction proteins in the mBECs, suggesting that multiple tight junction proteins are contributing to establishing barrier function in the mBECs (notably *Cldn 9, 7, 4, 6, 3, 12, 23* and *TJP 3*) (Fig. 4c).

**Fig. 5:**
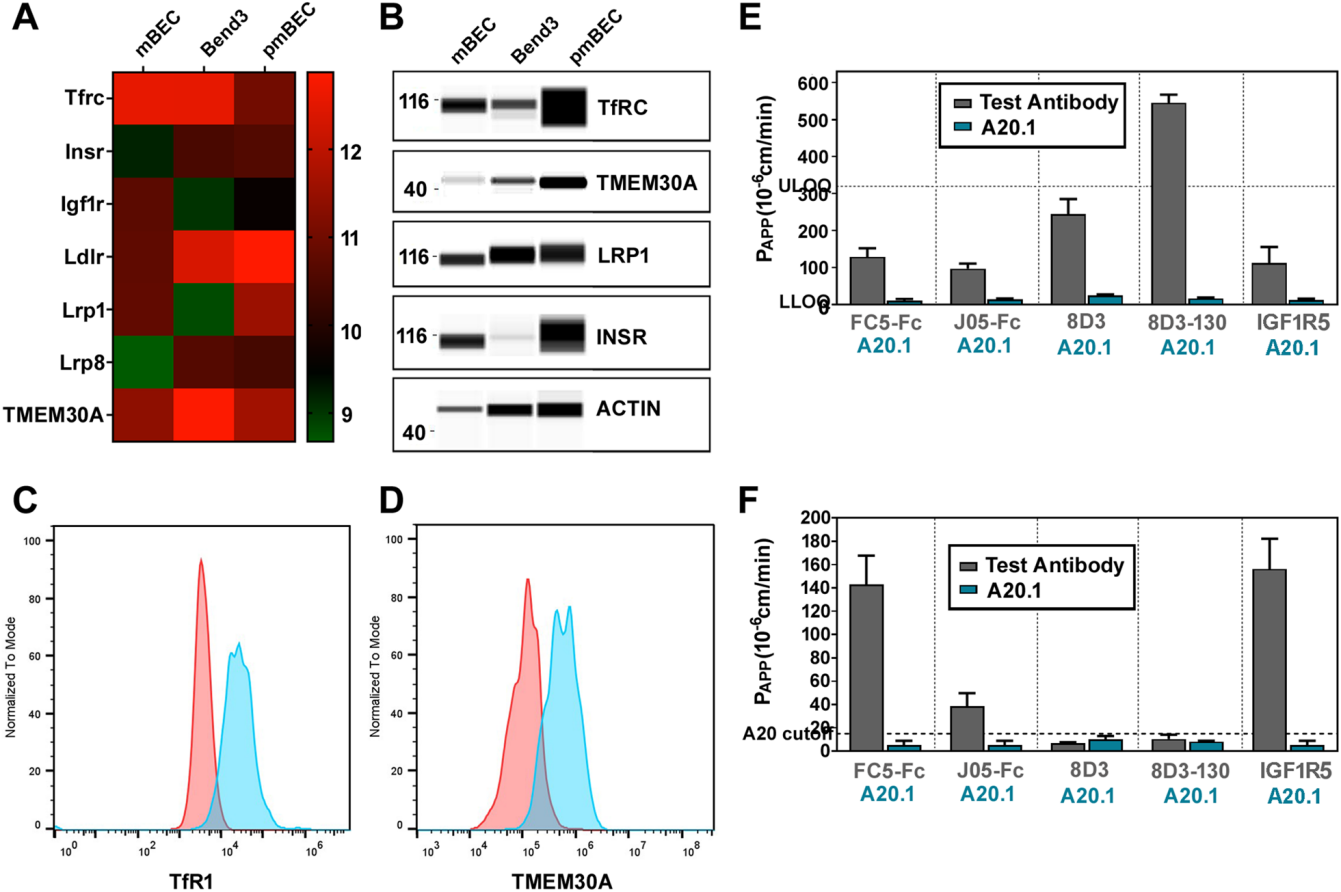
Functional receptor mediated transcytosis in mBECs. **A** RNASeq heatmaps depicting log2 transformed transcript abundances of expression of receptors involved in mediating receptor-mediated transcytosis (RMT) in mBEC, bEnd.3 and pmBEC. Green: low expression; Red: high expression. **B** Representative Wes blots confirming protein expression of key RMT receptors: TfRC, TMEM30A, LRP1, INSR; β-ACTIN was used as loading control. **C**, **D** Flow cytometry analysis assessing TfR1 and TMEM30A expression in mBECs. Red = unstained control. The in vitro apparent permeability coefficient (P_APP_) was assessed by MRM for TfR targeting antibodies J05-Fc, 8D3 and 8D3-130 as well as the species cross-reactive FC5-Fc (targeting TMEM30A) and IGF1R5-Fc (targeting IGF1R) in **E** the mouse mBEC and **F** and human iBEC BBB models. A20.1 (targeting *C. Difficile Toxin A*) was used as a negative transcytosis control antibody (mean ± SD). LLOQ (Lower limit of quantification); ULOQ (Upper limit of quantification). Results are from two independent differentiations

The mBECs also expressed a battery of solute carrier (*SLC*) transporters including those enriched at the BBB such as the glucose transporter *Glut-1* (*SLC2A1*) and *large neutral amino acid transporter-1* (*SLC7A5*) (Fig. 4d). The mBECs also expressed ATP-binding cassette (ABC) transporters (Additional file 1: Figure S6) that mediate efflux activity at the BBB including *P-gp* *(ABCB1*) and *breast cancer resistant protein* (*ABCG2*) as well as members of the multidrug resistance protein (MRP) family *ABCC1* (*MRP1*)*, ABCC4* (*MRP4*)*, and ABCC5* (*MRP5*) (Fig. 4d). Lastly, key RMT receptors and transporters such *transferrin receptor (TfR), Insulin receptor (Insr), Insulin-like growth factors (IGF1R), Low-density lipoprotein receptor (LDLR)-related protein 1 and 8 (LRP1, Lrp8) and Transmembrane protein 30A (TMEM30a)*, are also highly expressed in the mBECs (Fig. 5a), as further validated by Western blotting (Fig. 5b) and flow cytometry for TfR and TMEM30A (Fig. 5c).

The key differentially regulated pathways that were down-regulated in mBECs included ECM-receptor interactions and focal adhesions pathways (Table 2). The down-regulation of cytoskeletal, ECM and adherens junction genes could be attributed to the differences in culture protocols and ECM coatings used to culture the mBEC (Matrigel/Collagen IV/Fibronectin) versus pmBEC (Gelatin) and bEnd.3 (Collagen I) cells. One of the main down-regulated transcripts in mBECs was *Caveolin-1 (Cav-1)*, the principal component of caveolae, endocytic vesicles that provide a route for Cav-1 dependent endocytosis and potentially transcytosis. Caveolae are downregulated in mature BECs [60]and evidence suggests that BBB dysfunction, following injury or disease, is accompanied by increased levels of Cav-1 expression and increased BBB permeability [61, 62]. The pathways that were up-regulated in mBECs, compared to bEnd.3 and pmBECs, were related to cell cycle, DNA replication and spliceosome, reflecting the overall highly proliferative nature of the mBECs during the course of differentiation. Overall, this transcriptomic profile is very similar to what has been reported for human iPSC-derived BECs [48]; henceforth, we have revised the definition of mBECs to mouse brain endothelial-like cells.

**Table 2:** Differentially expressed genes in mBECs compared to pmBECs

Pathways up			Pathways down	
Cell cycle	DNA replication	Spliceosome	ECM receptor interaction	Focal adhesion
BUB1	DNA2	ALYREF	CD36	CAV1
BUB1B	LIG1	EIF4A3	CD44	CAV2
CCNA2	MCM2	HNRNPM	COL1A1	COL1A1
CCNB1	MCM3	LSM2	COL1A2	COL1A2
CCNB2	MCM4	LSM3	COL3A1	COL3A1
CCNE1	MCM5	LSM4	COL4A4	COL4A4
CDC20	MCM6	LSM5	COL4A6	COL4A6
CDC25C	MCM7	MAGOH	COL5A1	COL5A1
CDC45	PCNA	PPIH	COL5A2	COL5A2
CDC6	POLA1	PPIL1	COL5A3	COL5A3
CDC7	POLA2	PRPF3	COL6A1	COL6A1
CDK1	POLD1	RBM8A	COL6A2	COL6A2
CDKN1C	POLE	SNRNP40	COL6A3	COL6A3
CHEK1	POLE2	SNRPA1	COL6A6	COL6A6
CHEK2	PRIM1	SNRPD1	FN1	EGFR
DBF4	RFC2	SNRPE	ITGA1	FN1
E2F2	RFC3	SNRPG	ITGA11	HGF
ESPL1	RFC4	SRSF1	ITGA2	ITGA1
MAD2L1	RFC5	SRSF7	ITGA5	ITGA11
MCM2	RNASEH2B		ITGA7	ITGA2
MCM3	RPA1		ITGA8	ITGA5
MCM4	RPA2		ITGB3	ITGA7
MCM5	RPA3		ITGB4	ITGA8
MCM6			ITGB6	ITGB3
MCM7			ITGB8	ITGB4
ORC1			LAMA2	ITGB6
PCNA			LAMA3	ITGB8
PKMYT1			LAMA4	KDR
PLK1			LAMB3	LAMA2
PTTG1			RELN	LAMA3
SKP2			SV2B	LAMA4
SMC1B			SV2C	LAMB3
TKK			THBS1	MYLK
			THBS2	MYLK2
			TNC	PDGFD
			TNR	PDGFRB
			TNXB	PRKCA
			VWF	PRKCB
				RASGRF1
				RELN
				THBS1
				THBS2
				TNC
				TNR
				TNXB
				VWF

Differentially expressed genes were analyzed with Gene Set Enrichment Analysis (GSEA, version 4.0.3) using C2 Canonical Pathways gene sets database with 1000 permutations. Significantly changed pathways in mBECs were summarized in this table.

### Receptor mediated transcytosis in mBECs

Since the mBECs showed strong expression of RMT-specific receptors and transporters (Fig. 5a–d), we assessed functional RMT triggered by antibodies raised against these receptors. Specifically, we focused our studies on assessing the apparent permeability (P_APP_) of a panel of antibodies binding either species selective or cross-reactive epitopes on RMT receptors including TfR, TMEM30A and IGF1R (Table 1) in both mBECs and human iBEC BBB models. We compared the directional transendothelial transcytosis of a species cross-reactive camelid single-domain antibody (V_H_H) FC5, which targets TMEM30A, fused to (human or mouse) Fc fragment (FC5-Fc; 80 kDa) [11] and the species cross-reactive anti-IGF1R single-domain antibody (IGF1R5-Fc; 80 kDa) which targets IGF1R [67]. We also examined both species-cross reactive V_H_H targeting TfR (J05-Fc; 80 kDa) and mouse-specific TfR-binding IgG variants with different affinities to mouse TfR (8D3; 150 kDa) [6, 63, 64]. J05-Fc is camelid V_H_H isolated from the naïve pan-camelid V_H_H phage-display library by sequential panning against immobilized human and rat TfR extracellular domains, which also demonstrated cross-reactivity with mouse TfR (data not shown). The antibody has low affinity of 350 nM and 750 nM for rat and human TfR, respectively. In this study, it has been used in bi-valent fusion to N-terminus of human Fc (J05-Fc).

The 8D3 IgG variants included two different TfR-binding affinities (1.2 nM and 130 nM). All transcytosis assays also included a non-crossing control antibody raised against *C. difficile toxin A* (A20.1; 13 kDa) with no known mammalian target. The rate of transcytosis of the antibodies (measured by the apparent permeability coefficient, P_APP_) was quantified using highly sensitive multiplexed nanoLC-SRM, as previously described [11, 12, 65]. All transcytosis experiments were performed with TEER values within the range of 300–500 Ω cm^2^ for the mBEC and iBECs, which we have found to be sufficiently tight enough for assessing antibody triggered RMT in vitro [10].

The species cross-reactive antibodies, FC5-Fc, J05-Fc and IGF1R5-Fc, showed similar P_APP_ values in the mBEC BBB model of 129, 97 and 112 × 10^–6^ cm/min respectively (Fig. 5e). The mBEC model was also able to discriminate differences in BBB transcytosis among 8D3 IgGs affinity variants, where higher affinity 8D3 (1.2 nM) showed lower P_APP_ values (244 × 10^–6^ cm/min) compared to lower affinity 8D3 (130 nM; 545 × 10^–6^ cm/min) (Fig. 5e). By contrast, 8D3 IgGs (which do not recognize the human TfR), showed negligible transport in the human iBEC model (P_APP_ values < 6 × 10^–6^ cm/min) (Fig. 5f). In both models, the P_APP_ values for FC5-Fc and IGF1R5-Fc were very similar, validating species-cross reactivity, as previously reported [10, 66, 67]; however, P_APP_ values for J05-Fc were approximately 2.5-fold lower in the human (38 × 10^–6^ cm/min) compared to the mouse (97 × 10^–6^ cm/min) BBB model; this may have been due to either lower affinity of J05 to human TfR compared to mouse TfR, or to differences in TfR expression in the two models.

**Fig. 6:**
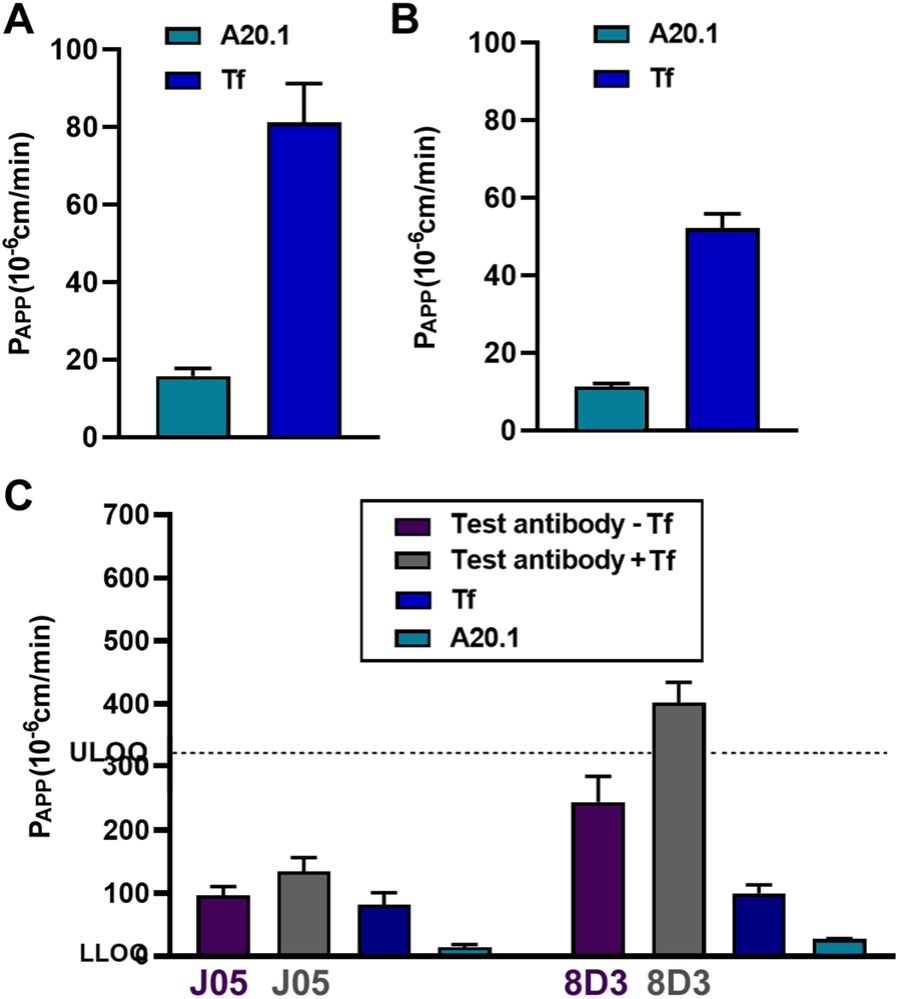
Species selectivity transport of Tf and TfR-targeting antibodies across the BBB. The in vitro apparent permeability coefficient (P_APP_) assessed by MRM of holo-transferrin (Tf) in **A** the mouse mBEC and **B** human iBEC BBB models (mean ± SD). **C** Comparison of the in vitro apparent permeability coefficient (P_APP_) for TfR targeting test antibodies J05-Fc, 8D3 (1.2 nM) in the presence or absence of 2 mg/ml of Tf in the luminal chamber in the mouse mBEC model (mean ± SD). A20.1 (targeting *C. Difficile Toxin A*) was used as a negative transcytosis control antibody (mean ± SD). LLOQ (Lower limit of quantification); ULOQ (Upper limit of quantification). Results are from two independent differentiations

We also assessed transport of the anti-TfR antibodies (J05-Fc and 8D3) in the presence and absence of the natural ligand holo-transferrin (Tf), at physiological plasma concentrations (2 mg/ml) [68], to evaluate whether these antibodies competitively inhibited holo-Tf binding and transcytosis. P_APP_ values for holo-Tf transport were approximately 80 × 10^–6^ cm/min in the mBEC model (Fig. 6a) and 52 × 10^–6^ cm/min in the iBEC model (Fig. 6b). These results highlight possible species differences in TfR expression supporting literature data on higher TfR expression levels in mouse BEC [1]. The addition of holo-Tf, together with the 8D3 (1.2 nM) or J05-Fc antibodies, increased the P_APP_ values for high affinity 8D3 antibody (244 vs 402 × 10^–6^ cm/min) in the mBEC model, whereas no significant changes in the J05-Fc P_APP_ values were observed (Fig. 6c) The studies suggested that the J05 antibody epitope binding sites were not competing or interfering with the binding and internalization of the natural ligand. The enhancement of transcytosis of 8D3 in the presence of Tf may indicate that the conformational change in TfR caused by Tf binding may affect the nature of 8D3 binding (e.g., affinity) to its epitope that facilitates transcytosis.

These RMT studies demonstrated the ability of stem cell derived human and mouse BBB models to discern species-unique attributes of a panel of mouse TfR antibodies. Collectively, these experiments highlight the requirement of translational BBB models to discriminate species specificity and selectivity of antibodies.

## Discussion

Stem cell derived BBB models in vitro are useful for advancing the understanding of the BBB development and dysfunction in disease, as well as for the screening and evaluation of novel CNS targeting therapeutics. Species differences in RMT receptor expression and abundance in BEC [1, 3, 5] and the species selectivity of antibody-based BBB-targeting carriers [6], necessitate the development of translational BBB models from different species.

In this study, we have used mESC-D3 to develop a mouse derived BBB-model. The mESC-D3 cells represent a renewable and scalable cell source for the efficient derivation of mBECs as well as other syngeneic cells of the NVU. We developed a directed monolayer differentiation strategy, recapitulating the developmentally-relevant progression from mesodermal to endothelial progenitor lineages, yielding a pure population of mBECs following matrix selection. The mBECs exhibit many elements of the molecular and functional phenotype of mouse BECs such as high TEER values, BBB-specific gene/protein expression profile, functional polarized BBB transport and species-specific properties. However, the mBEC also display some epithelial-like characteristics, similar to their human iPSC-derived BECs counterparts [35, 48, 53], such as cobblestone vs spindle like morphology and epithelial and choroid plexus-like transcriptomic profiles. Although the mBECs formed vascular tube-like structures in a 3D Matrigel angiogenesis assay, it is well documented that different cell types can form tubes in Matrigel hence a more stringent measure of angiogenic potential would be to employ the Fibrin bead assay [69]. Nevertheless, in vitro models using these cells typically exhibit strong functional barrier properties and also express multiple BBB specific receptors, transporters and efflux pumps; important criteria for studying barrier regulation and drug delivery applications in the CNS. We have shown that the mBECs are a useful model for use in ‘translational’ screening of early CNS targeting pipelines, especially those targeting RMT. To our knowledge, this is the first described method for differentiating mESC-D3 into mBECs with functional barrier properties circumventing the scalability and weakened barrier properties of primary and immortalized mouse BECs.

Compared to previously described mouse ESC-derived endothelial cells protocols [23, 24, 70–74], we adapted the differentiation strategy to generate brain endothelial-like cells with barrier properties ranging between 350–400 Ω cm^2^ in the presence of ACM, RA and hydrocortisone, collectively known to provide instructive cues inducing barrier tightening [10, 31, 36, 37, 39]. In fact, the nuclear retinoid X receptor α (RXRα) signaling cascade has been shown to be specifically enriched at the BBB, implicating this pathway in regulating this vital barrier [54]. These TEER values translate into low sodium fluorescein permeability (Pe = 1.7 × 10^–5^ cm/min) for the mBECs, very similar to that observed in our human iBEC model (Pe = 2.0 × 10^–5^ cm/min) and substantially lower than that in bEnd.3 (Pe = 1.02 × 10^–3^ cm/min) and SV-ARBEC immortalized BBB lines (Pe = 0.44 × 10^–3^ cm/min). A number of reports have demonstrated a synergistic effect of RA and the NVU co-culture models of pericytes, astrocytes and neurons resulting in iPSC-derived BEC TEER values between 3 000 to 25 000 Ω cm^2^ (reviewed in [4]). Although we have shown that the mBECs show a tightening response to astrocytic cues, the potential synergies among co-culture with cells from the NVU and chemical (RA) induction remain to be fully examined in our mBEC model.

Transcriptomic analysis of the mBECs also highlighted that the barrier formation in the mBECs is driven by the expression of a number of different tight junction proteins. Notably, we observed that the mBECs express high levels of *Cldn 3, 4, 6, 7, 9, 23* and *TJP 3*. Of these, *Cldn 3,4, 5* and *7* are known to be sealing Claudins [75, 76]; whereas *Cldn 4* and *6* are specifically related to epithelial lineages—which may be collectively contributing to forming and stabilizing tight junctional complexes thereby inducing TEER formation and restricting paracellular permeability in the mBECs. *Cldn3* has been shown to be expressed primarily at the blood-cerebrospinal fluid barrier and not specifically at the BBB [77]. Furthermore, although transcript levels of *Cldn5* in the mBECs were lower than in pmBEC and bEnd.3 cells, we observed high protein expression levels by flow cytometry with continuous membrane expression between adjacent cells, as assessed by immunostaining. Similar to our observations, Girard et al. also described discrepancies between mRNA expression levels and protein detection of Cldn 5 in iPSC-derived BECs and human primary BECs despite confirming antibody specificity for Cldn 5 [35]. These observations highlight the importance of validating cell structure and function in addition to global transcriptomic profiling results. Although, the discrepancies between Cldn 5 mRNA and protein expression remain unexplained, it may be related to low protein turn-over of stably expressed proteins. In fact, inhibition of GSK3β in human BECs has been shown to lead to a decrease in Cldn 5 and Occludin protein turnover not transcriptional regulation [78]. Whether a similar phenomenon is happening in the mBECs would require further investigation.

The mBECs were also found to have lower expression of canonical endothelial markers (CD31, KDR, Angpt2, Cdh5) compared to pmBECs and bEnd.3 cells. This has also been observed in other mouse ESC-derived endothelial cells, independent of BBB phenotype, and has been attributed to a lack of complete functional maturation in vitro [49]. While it is true that BEC exhibit the properties of the BBB, these properties are not intrinsic to the endothelial cells but are induced by the interactions with the CNS microenvironment [79]. In this protocol, we differentiated endothelial cells from mesodermal/endothelial progenitors and used ACM to provide the neural instructive cues to induce BBB-specific phenotype. Although this was sufficient to induce barrier formation, incorporation of other modulators of vascular properties such as hypoxia [80], shear stress [52, 81, 82], three dimensional architecture [81] and neural cells (neurons, astrocytes and pericytes) would more closely recapitulate the microenvironment required for the induction and maintenance of the BBB phenotype in the mBECs.

BECs are characterized by a decreased number of endocytic/pinocytic vesicles compared with endothelial cells in peripheral tissues, which greatly limits the transcellular movements of hydrophilic molecules between the blood and the brain. An increase in the number of intracellular vesicles in BECs has been observed in several diseases in which there is a breakdown of the BBB [60, 83, 84]. Interestingly, one of the most down-regulated transcripts in the mBECs was *Caveolin-1* (*Cav-1*), the principal component of caveolae, endocytic vesicles that provide a route for Cav-1-dependent endocytosis and potentially transcytosis. Coincidently, Cav-1 was also observed as the most down-regulated transcript in the iBECs [9]. Caveolae are known to be downregulated in mature BECs [60]; whereas, BBB dysfunction, following injury or disease, is accompanied by increased levels of Cav-1 expression and increased BBB permeability [85]. For example, increased bulk-transcytosis of circulatory albumin across the BBB has been observed in Mfsda2 knock-out animals which exhibit up-regulation of caveolae in BECs [86]. In addition, PLVAP, a transmembrane protein associated with the caveolae of fenestrated microvascular ECs [51], was also down-regulated in mBECs. In mice, PLVAP expression is enriched in non-CNS endothelial compared to CNS endothelial cells [54]. PLVAP expression in BECs only occurs in pathological conditions associated with a compromised barrier function such as cancer, ischemic stroke and diabetic retinopathy. As such, decreased vesicular transport proteins Cav-1 and PLVAP may be good biomarkers of barrier maturation in cultures BECs.

In addition, the specific RMT receptor abundance can impact the transcytosis of their ligands, and has been shown to differ significantly among species. For example, TfR is threefold more abundant in rodent compared to human brain vessels [1, 3, 87, 88], underscoring the importance of species relevant BBB models that replicate in vivo species differences. In agreement with this literature evidence, we were able to demonstrate that the transcytosis rates of holo-transferrin, a natural ligand for TfR, is significantly lower in the human iBEC model compared to the mouse mBEC models. To explore the utility of the mBEC model in the evaluation of RMT of engineered antibody ligands, we assessed a panel of TfR-targeted antibodies with different species cross-reactivity and varying affinities. Two BBB models in vitro, human iBEC and mouse mBECs, were able to reliably discriminate species-selective or cross-reactive TfR antibodies; mouse-selective 8D3 antibody showed highly facilitated transcytosis in mBEC model and no BBB crossing in iBEC model; whereas a cross-reactive J05 showed similar levels of enhanced transcytosis in both models. Furthermore, two other species cross-reactive antibodies against different RMT receptors, TMEM30A (FC5) and IGF1R (IGF1R5) exhibited similar enhanced transcytosis in both BBB models. The mBEC model also reproduced reliably in vivo findings of improvement in transcytosis of TfR antibody 8D3 when its binding affinity to TfR was reduced from 1 to 130 nM [89, 90]. Another interesting observation in this study was the ability of holo-transferrin to increase the rate of transcytosis of the high-affinity 8D3 antibody, whereas it did not affect transcytosis of either low-affinity 8D3 or J05 antibodies. Although the exact mechanism of this effect is not clear, it may be plausible that holo-transferrin may stimulate endocytosis and receptor recycling allowing more efficient transport/release of the high affinity 8D3 antibody.

As demonstrated in this study, coupling evaluation of RMT-targeting antibodies in rodent (mouse or rat) and human BBB models can be an important strategy for identifying species cross-reactive antibodies and assessing similarly efficient antibody variants in pre-clinical in vivo brain distribution and pharmacokinetic studies. Since the mouse is the most widely used pre-clinical animal model for disease modeling, discovery and evaluation of brain delivery ‘shuttles’, mouse BBB models in vitro can be more predictive of quantitative in vitro/in vivo pharmacokinetic correlations. Although the mBEC model is a valuable tool to assess species cross-reactive antibodies, advancing more effective CNS targeting therapies will ultimately rely on the validation of human binders in human BBB models. Nevertheless, mESC-derived mBECs can serve as part of a translational suite of BBB assays to assess CNS targeting biotherapeutics, small molecules and CNS-tropic viruses.

However, similar to their human iPSC-BEC counterparts [48, 52, 53], the mBECs have similar limitations since they display a more epithelial-like phenotype. To improve the fidelity of BEC differentiation, several human iPSC-based BEC protocols have been reported with significant differences in barrier properties; those with robust endothelial features display high permeability whereas those with high barrier properties show principally epithelial-like features [35]. Collectively, the growing body of evidence suggests that stem cell derived BECs share some key characteristics of “mature” endothelial cells, while retaining some markers of alternative phenotypes and immature endothelium due to limited functional maturation in vitro. These observations suggest that the BEC differentiation protocols may be missing an important signal for complete functional differentiation and maturation in vitro and further optimization of differentiation techniques is still required. As such, it is prudent to exercise caution when utilizing these cells for studies where the endothelial phenotype is crucial [91]. Since all in vitro models have limitations and divergence from their in vivo cellular counterparts, these model predictions should always be validated and tested in complementary in vitro and in vivo assays.

## Supplementary Information

**Additional file 1: Fig. S1–S6.**

**Additional file 2: Table S1. **Peptide ‘signature’ used for nanoLC-SRM analysis. **Table S2: **Antibodies used for Immunofluorescence, WES and Flow Cytometry. **Table S3: **RT-PCR Primers

## Data Availability

The datasets generated during and/or analyzed during the currenponding author upon request.
