# Supplementary material for: Mouse embryonic stem cell-derived blood–brain barrier model: applicability to studying antibody triggered receptor mediated transcytosis
Source: Fluids Barriers CNS. 2023 May 26;20:36. doi: 10.1186/s12987-023-00437-0 (PMC10224255; doi:10.1186/s12987-023-00437-0)
Supplement: Supplementary file 1 — Additional file 1: Fig. S1–S6. [file 12987_2023_437_MOESM1_ESM.pdf]

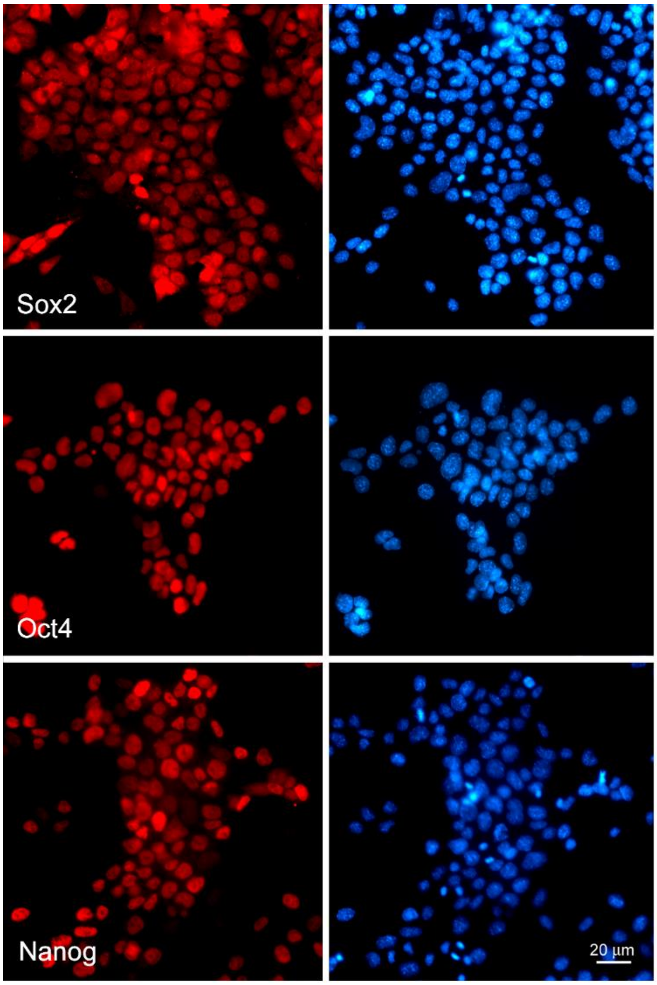

Supplementary Figure 1: mESC-D3 subculture on Matrigel coated plates in mES medium. The mESC-D3 cells sub-cultured and adapted on Matrigel coated plates in the presence of mES medium retained expression of key pluripotency markers Sox2, Oct4 and Nanog (all red); Hoechst counterstain (blue). Scale bar = 20 µm

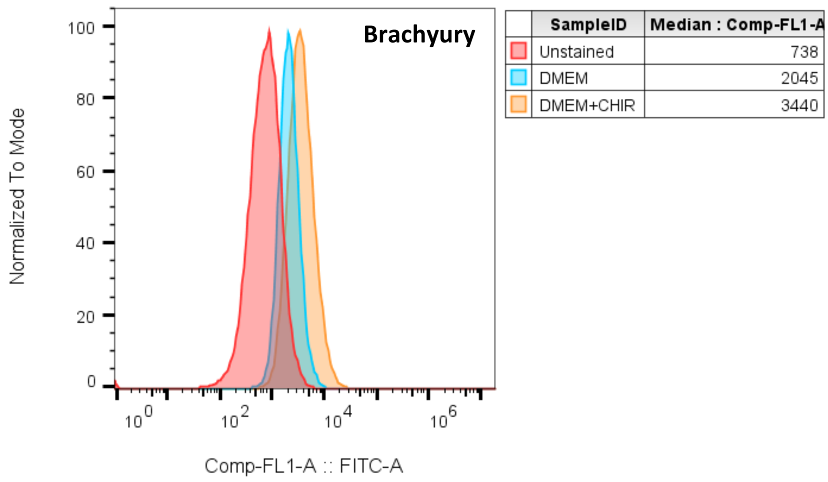

	SampleID	Median : Comp-FL1-A
	Unstained	738
	DMEM	2045
	DMEM+CHIR	3440

Supplementary Figure 2: Induction of mesoderm in the presence of Wnt agonist CHIR99021. Treatment with CHIR99021 during the first day of mesoderm induction increased the number of Brachyury positive cells. Red: unstained control; blue: DMEM mesoderm induction media without CHIR99021 and orange: DMEM mesoderm induction media supplemented with 1.4 mM CHIR99021 (CHIR). MFI values shown in insert table.

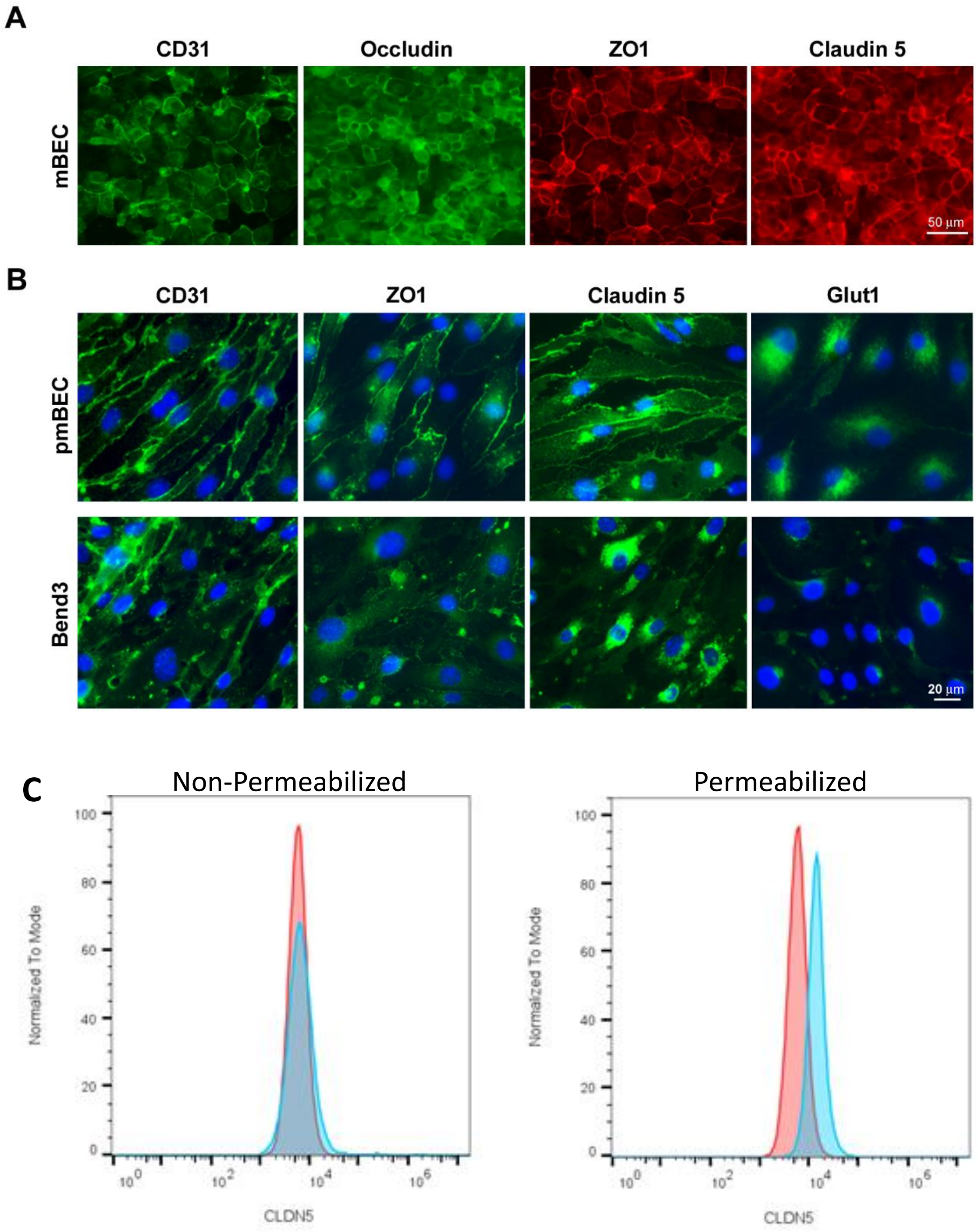

Supplementary Figure 3: Immunofluorescence comparison of mBEC, pmBEC and Bend3 cells. Immunofluorescence staining for endothelial (CD31, green) and tight junction markers (Occludin, ZO1 and Claudin 5 – all green) in mBEC (**A**) as well as (**B**) pmBEC and bEnd.3 cells. Hoechst counterstain (blue). Scale bar = 50 and 20 µm. (**C**) Flow cytometry assessment of Claudin 5 expression in non=permeabilized and permeabilized Hela cells. Red = unstained; Blue = stained.

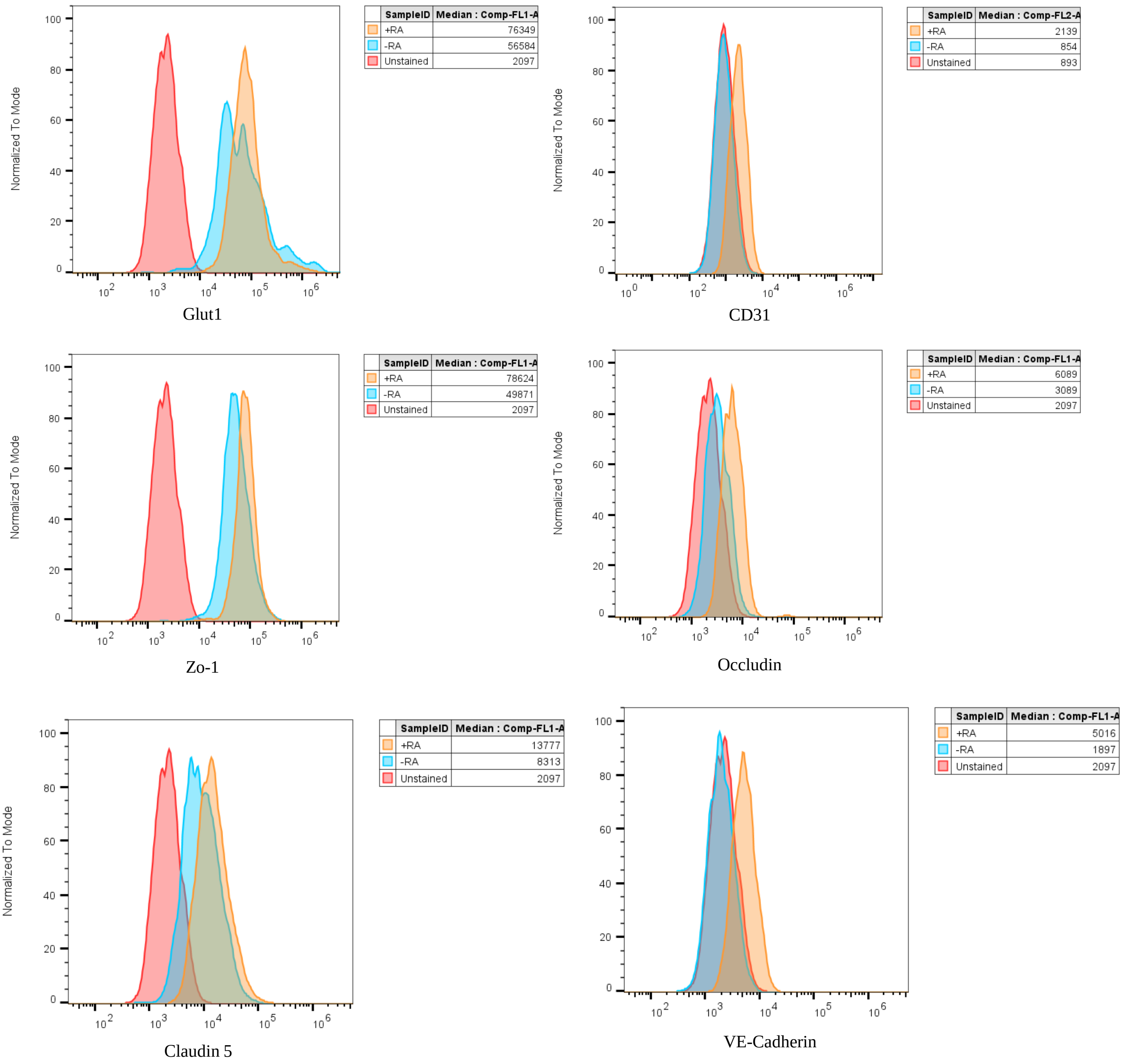

Supplementary Figure 4: Flow cytometry analysis for endothelial and BBB specific markers in the presence and absence of RA. Flow cytometry analysis for a panel of BBB and endothelial specific markers (GLUT1, CD31, ZO-1, OCCLUDIN, CLAUDIN5 and VE-Cadherin) in mBECs in the presence and absence of RA at day 8 of differentiation. Red: Unstained, Orange: 10 µM RA treatment and Blue: no RA treatment. MFI values shown in insert table. Results are of 2 independent differentiations.

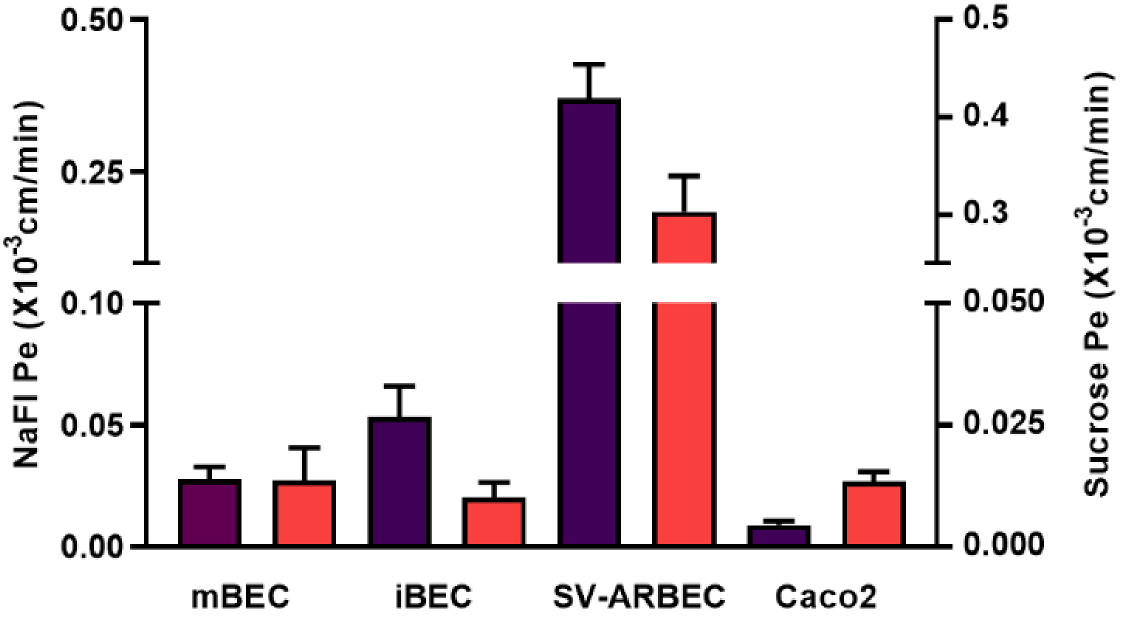

Supplementary Figure 5. Comparison of NaFl and Sucrose permeability. Comparison between sodium fluorescein (NaFl) (left y-axis) and sucrose permeability coefficient (Pe, right y-axis) in mBECs, human iPSC-derived BECs (iBECs), SV-ARBECs and Caco2 cells (mean ± SD). Results are of 3 independent differentiations.

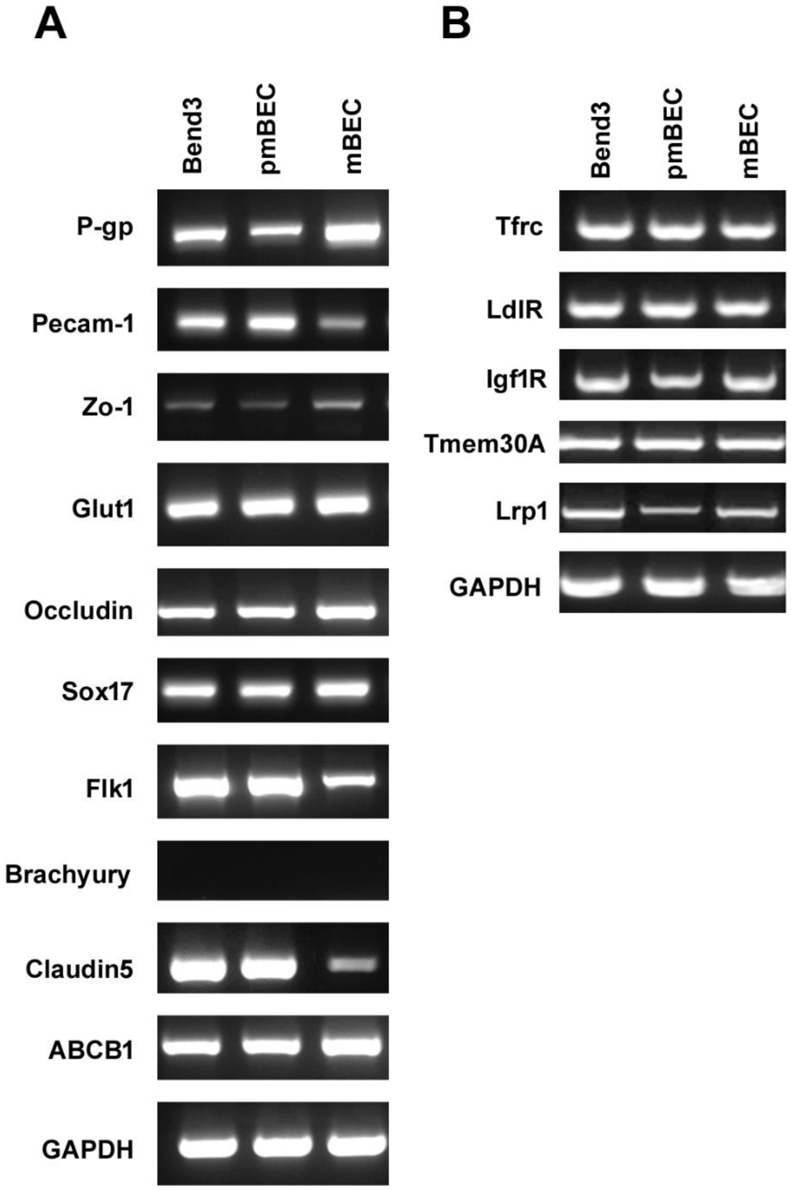

Supplementary Figure 6. RT-PCR for gene expression profiles. (**A**) Representative images of RT-PCR detection of endothelial, tight junction and (**B**) receptor mediated transport (RMT) receptor transcripts including in bEnd.3 , pmBEC and mBECs. Cropped gels shown. Methods described in Supplementary Methods.

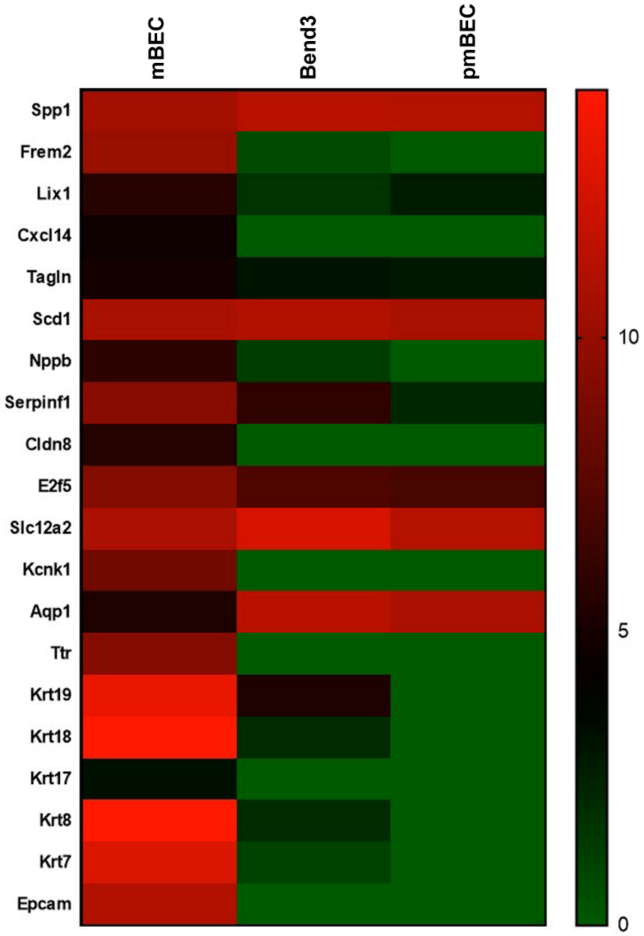

Supplementary Figure 7. Comparative RNASeq analysis of choroid plexus genes expression profiles in mBEC, bEnd.3 and pmBECs. Heatmaps depicting log2 transformed transcript abundances of choroid plexus epithelial gene expression profiles in mBEC, bEnd.3 and pmBECs. Green= low expression; Red= high expression.
