# Supplementary material for: Mouse embryonic stem cell-derived blood–brain barrier model: applicability to studying antibody triggered receptor mediated transcytosis
Source: Fluids Barriers CNS. 2023 May 26;20:36. doi: 10.1186/s12987-023-00437-0 (PMC10224255; doi:10.1186/s12987-023-00437-0)
Supplement: Supplementary file 2 — Additional file 2: Table S1. Peptide ‘signature’ used for nanoLC-SRM analysis. Table S2: Antibodies used for Immunofluorescence, WES and Flow Cytometry. Table S3: RT-PCR Primers [file 12987_2023_437_MOESM2_ESM.docx]

**Additional file 2**

**Additional file 2: Table S1. Peptide ‘signature’ used for nanoLC-SRM analysis**

| Protein | Type | Peptide Sequence | Charge | m/z of  peptide | Peptide fragment signatures (m/z) used for quantification |
| --- | --- | --- | --- | --- | --- |
| FC5-Fc | VHH (llama naïve) fused to N-terminus of human Fc from IgG1 | ITWGGDNTFYSNSVK | 2 | 844.92 | 534.48, 729.47, 737.89, 1288.44 |
|  |  | ITWGGDNTFYSNSVK^(a)^ | 2 | 848.92 | 542.48, 733.48, 741.87, 1296.44 |
|  |  | TTPPVLDSDGSFFLYSK | 2 | 937.28 | 836.8, 1150.24, 1265.44, 1378.72, 1477.44 |
|  |  | TTPPVLDSDGSFFLYSK^(a)^ | 2 | 941.28 | 840.5, 1158.25, 1273.44, 1386.74, 1485.44 |
| A20.1 | VHH, llama immune (C. diff toxin A) | TFSMDPMAWFR | 2 | 694.77 | 582.22, 807.4, 922.42, 1140.5 |
|  |  | EFVAAGSSTGR | 2 | 541.27 | 564.27, 635.31, 706.35 |
|  |  | EFVAAGSSTGR^(a)^ | 2 | 546.27 | 574.28, 645.32, 716.36 |
| J05-Fc | VHH (llama naïve) fused to Fc from IgG1 | LEESGGGLVQPGGSLR | 2 | 778.41 | 714.39, 586.33, 970.48 |
|  |  | DLPAPIER | 2 | 455.75 | 682.39, 341.70, 229.12 |
|  |  | APQVYVLPPPEEEMTK | 2 | 914.46 | 1170.57, 1057.49, 771.44 |
|  |  | NTEPVLDSDGSYFMYSK | 2 | 976.93 | 1608.72, 1299.52, 804.87 |
| 8D3 or  8D3-130 | IgG1 | TTPPVLDSDGSFFLYSK | 2 | 937.28 | 836.8, 1150.24, 1265.44, 1378.72, 1477.44 |
|  |  | TTPPVLDSDGSFFLYSK^(a)^ | 2 | 941.28 | 840.5, 1158.25, 1273.44, 1386.74, 1485.44 |
| Tf | Protein | SVIPSDGPSVACVK | 2 | 708.36 | 1116.54, 615.31, 558.77 |
| IGF1R5-Fc | VHH (llama naïve) fused to N-terminus of human Fc from IgG1 | TIDNYAMAWSRQ | 2 | 664.27 | 1113.52, 721.41, 448.29, 519.33 |
|  |  | LEESGGGLVQAGGSLR | 2 | 765.35 | 688.42, 787.42, 560.25, 489.33 |

* Shown are the transitions for nanoLC-SRM detection/quantitation of various V_H_Hs, and V_H_H-Fc fusion used in the study. In various experiments, analyses were multiplexed in different combinations for simultaneous monitoring of multiple peptides in the same sample.

**Additional file 2: Table S2: Antibodies used for Immunofluorescence, WES and Flow Cytometry**

| **Antibody** | **Supplier and Cat. no** | **Dilution** |
| --- | --- | --- |
| **Immunofluorescence Antibodies** | | |
| CD31 | R&D Systems (AF3628) | 1:100 |
| OCCLUDIN* | Invitrogen (710192) | 1:100 |
| ZO1* | Invitrogen (402200) | 1:100 |
| CLAUDIN 5* | Invitrogen (352588) | 1:50 |
| NANOG | Abcam (AB80892) | 1:200 |
| SOX2 | In house hybridoma | 1:100 |
| OCT3/4 | Abcam (AB19857) | 1:100 |
| TRANSFERRIN | Invitrogen (13-6800) | 1:200 |
| **Wes Antibodies** | | |
| TfR | Invitrogen (1306800) | 1:10 |
| TMEM30A | Abcam (Ab105062) | 1:10 |
| LRP1 | Abcam (Ab92544) | 1:200 |
| INSR | Cell Signaling (3025T) | 1:100 |
| ACTIN-HRP | Sigma (A3854) | 1:200 |
| **Flow Antibodies** | | |
| Brachyury | R&D Systems (IC2085G) | 1:100 |
| Flk1 | BD Biosciences (555308) | 1:100 |
| GLUT1 | Abcam (195359) | 1:100 |
| CD31 | Abcam (215911) | 1:100 |
| VCAM1 | BD Biosciences (561612) | 1:100 |
| VE-CADHERIN | BD Biosciences (562242) | 1:100 |
| TRANSFERRIN | BD Biosciences (567206) | 1:100 |
| TMEM30A | Bioss (BS16576R) | 1:100 |

* denotes antibodies that were used for immunofluorescence and flow cytometry

**Additional file 2: Table S3: RT-PCR Primers**

| **Gene** | **Sequence, from 5' to 3'** | **Amplicon** | **TM** | **Source** |
| --- | --- | --- | --- | --- |
| ABCB1-F | AGTGTCCACAGAAAGCAAGACCA | 357 bp | 55^o^C | Leukemia 27  2013  p. 32-40 |
| ABCB1-R | TCGGACTCGCTTGGTGAGGATCT |  |  |  |
| CLDN5-F | TGGAACGCTCAGATTTCATC | 292 bp | 55^o^C | Scandinavian J.Immuno  75, 2012  p.588-598 |
| CLDN5-R | AGGAAGGCAACCCCTCTAAG |  |  |  |
| ZO1-F | GCTAAGAGCACAGCAATG GA | 245 bp | 60^o^C | Mol. Biol. of Cell 19 2008 p.2465-2475 |
| ZO1-R | GCATGTTCAACGTTATCCAT |  |  |  |
| Glut1- F | GCTGTGCTTATGGGCTTCTC | 114 bp | 60^o^C | Blood 112 (12) 2008 p.4729-4738 |
| Glut1- R | CACATACATGGGCACAAAGC |  |  |  |
| PECAM 1-F | GAGCCCAATCACGTTTCAGTTT | 118 bp | 60^o^C | Amer. J. Path 161 (1) 2002 p.35-41 |
| PECAM 1-R | TCCTTCCTGCTTCTTGCTAGCT |  |  |  |
| Occludin-F | AGTACATGGCTGCTGCTGATG | 372 bp | 60^o^C | In-House Design  NCBI Primer Blast |
| Occludin-R | AATTGGAGTGTTCAGCCCAGT |  |  |  |
| Brachyury-F | CTGGGAGCTCAGTTCTTTCG | 176 bp | 60^o^C | BMC Biol 12 (63) 2014  p. 1-19 |
| Brachyury-R | GTCCACGAGGCTATGAGGAG |  |  |  |
| FLK1-F | TTGGAGCATCTCATCTGTTACAGC | 695 bp | 60^o^C | PNAS 102 (4) 2005 p. 1076-1081 |
| FLK1-R | GGCCGGCTCTTTCGCTTACT |  |  |  |
| SOX17-F | ATACGCCAGTGACGACCAGAG | 126 bp | 60^o^C | Nature Comm  5 (4000)  2014 p. 1-12 |
| SOX17-R | ACCACCTCGCCTTTCACCTTTA |  |  |  |
| GAPDH-F | ACCACAGTCCATGCCATCAC | 452 bp | 60^o^C | In-House Design  NCBI Primer Blast |
| GAPDH-R | TCCACCACCCTGTTGCTGTA |  |  |  |
| TfR-F | AAAGTTTCTGCCAGGCCCTTA | 291 bp | 60^o^C | In-House Design  NCBI Primer Blast |
| TfR-R | CGCTGCTGTACGAACCATTTG |  |  |  |
| LDL-F | TTTGGAGGATGAGAACCGGC | 250 bp | 60^o^C | In-House Design  NCBI Primer Blast |
| LDL-R | CAGGTACTGGCAACCACCAT |  |  |  |
| IGF1R-F | GCCTCCAACTTCGTCTTTGC | 857 bp | 60^o^C | In-House Design  NCBI Primer Blast |
| IGF1R-R | TTATTCTGCTCCACTTCTGGC |  |  |  |
| TMEM30A-F | CCTGGAAAAGAGCCTCGAA | 279 bp | 60^o^C | In-House Design  NCBI Primer Blast |
| TMEM30A-R | CCGTTTCCGTCCATACAAAGG |  |  |  |
| LRP1-F | GAGTGTCCGCATAGATGCCA | 996 bp | 60^o^C | In-House Design  NCBI Primer Blast |
| LRP1-R | TTATCGCCTGTGTAACGGGG |  |  |  |

**Supplementary Methods**

### RNA extraction and RT-PCR

Total RNA was extracted from cells, using TriReagent RT (Molecular Research Centre), as per manufacturer’s instructions. The RNA was treated with DNase (Turbo DNA-Free Kit, Ambion) to remove any residual DNA contamination and the RNA concentration was quantified using a NanoDrop ND-100 (Thermo Scientific). cDNA was synthesized using 5–10 µg of RNA using Superscript II Reverse Transcriptase (Life Technologies) and AncT primers (Life Technologies) and purified using the QiaQuick PCR purification kit (Qiagen). The Oligreen Assay (Molecular Probes) was used to measure the concentration of cDNA samples. For each RT-PCR reaction, 10 ng of cDNA, 10 pmol/µl primer sets specific for the genes of interest (Supplementary Table 3 ) and iQ Supermix (BioRad) were used and the PCR reaction was carried out in a PTC-200 DNA Engine Thermal Cycler (MJ Research) by using the following parameters: Initial denaturation step at 94°C for 3 min, followed by 30 cycles of a denaturation step at 94°C for 20 sec, annealing at 60°C for 20 sec and extension at 72°C for 20 sec, and a final extension step at 72°C for 3 min. RT-PCR reactions were run on 2% agarose gel and images detected using a Fluochem 8900 imager (Alpha Innotech).
